# Identification of potential target genes and crucial pathways in small cell lung cancer based on bioinformatic strategy and human samples

**DOI:** 10.1371/journal.pone.0242194

**Published:** 2020-11-13

**Authors:** Xiuwen Chen, Li Wang, Xiaomin Su, Sen-yuan Luo, Xianbin Tang, Yugang Huang

**Affiliations:** 1 Department of Pathology, Taihe Hospital, Hubei University of Medicine, Hubei, China; 2 Department of Immunology, Nankai University School of Medicine, Tianjin, China; Virginia Commonwealth University, UNITED STATES

## Abstract

Small cell lung cancer (SCLC) is a carcinoma of the lungs with strong invasion, poor prognosis and resistant to multiple chemotherapeutic drugs. It has posed severe challenges for the effective treatment of lung cancer. Therefore, searching for genes related to the development and prognosis of SCLC and uncovering their underlying molecular mechanisms are urgent problems to be resolved. This study is aimed at exploring the potential pathogenic and prognostic crucial genes and key pathways of SCLC via bioinformatic analysis of public datasets. Firstly, 117 SCLC samples and 51 normal lung samples were collected and analyzed from three gene expression datasets. Then, 102 up-regulated and 106 down-regulated differentially expressed genes (DEGs) were observed. And then, functional annotation and pathway enrichment analyzes of DEGs was performed utilizing the FunRich. The protein-protein interaction (PPI) network of the DEGs was constructed through the STRING website, visualized by Cytoscape. Finally, the expression levels of eight hub genes were confirmed in Oncomine database and human samples from SCLC patients. It showed that *CDC20*, *BUB1*, *TOP2A*, *RRM2*, *CCNA2*, *UBE2C*, *MAD2L1*, and *BUB1B* were upregulated in SCLC tissues compared to paired adjacent non-cancerous tissues. These suggested that eight hub genes might be viewed as new biomarkers for prognosis of SCLC or to guide individualized medication for the therapy of SCLC.

## Introduction

Lung cancer is a subtype of malignant tumors with a peak risk of morbidity and mortality, which makes it a notable healthcare issue for human beings. In 2018, the total number of cases newly diagnosed as lung cancer was about 2.09 million (11.6% of all newly diagnosed cancers), and the number of new deaths was about 1.76 million (18.4% of all sites) worldwide [1]. Small cell lung cancer (SCLC) accounts for approximately 20% of lung cancer patients and belongs to neuroendocrine tumor [2]. Different from other histopathological subtypes of lung cancer, SCLC is accompanied by rapid clinical progression. Almost all patients with SCLC have extensive metastasis when diagnosed, and it has a poor prognosis. The 5-year relative overall survival (OS) rate is not more than 6%. In clinical practice, conventional treatments of SCLC include chemotherapy, radiotherapy, surgery and immunotherapy. Chemotherapy for SCLC is the main treatment currently, but there are still many problems such as drug resistance and easy relapse [3, 4]. In the past few decades, the survival rate of SCLC patients has not been evidently improved, and no molecular-targeted drugs have been shown to significantly prolong the survival time of patients [5]. In recent years, studies have been reported that many molecular mechanisms altered in SCLC including induced expression of oncogene, such as *MYC* [6] and *FGFR1* [7], and deletion of tumor-suppressor genes, such as *TP53*, *PTEN*, *RB*, and *FHIT* [8]. Changes in these related genes and signaling pathways promote cell proliferation and inhibition of apoptosis, leading to early-stage metastasis of tumor cells, such as mutation, methylation or expression of *PIK3CA*, *PTEN*, *AKT* and other genes in the *PI3K/AKT/mTOR* pathway [9].

Because of the complexity of biological characteristics and poor prognosis of SCLC, the key biomarkers and specific targets for occurrence and development of SCLC are not well known. Therefore, it is necessary to explore more genetic information to screen out potential or promising biomarkers for early-stage diagnosis and precision medical treatment of SCLC. In recent years, gene chip technology and bioinformatics analysis has been widely used to identify molecular changes in tumorigenesis and development and has been proved to be an efficient method for identifying key genes in the research of genomics [10–12]. However, due to the strong invasion and short life span of SCLC, the data of related gene chips are infrequent.

**Fig 1:**
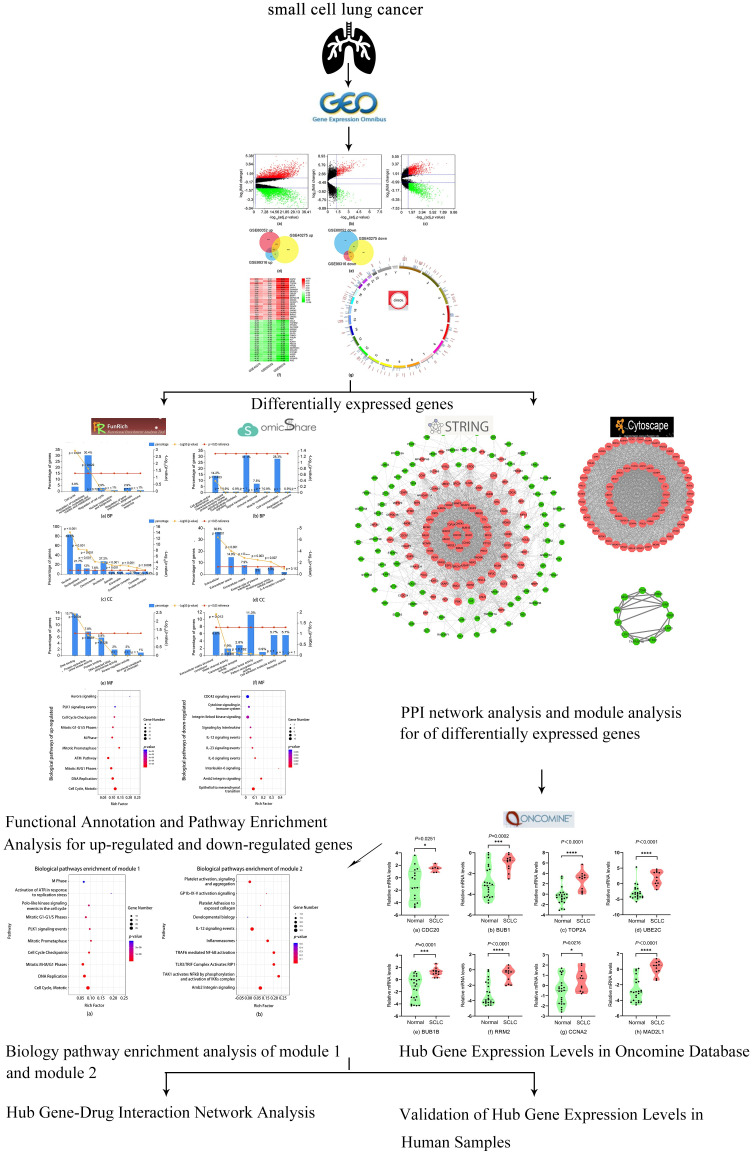
Workflow for bioinformatics strategy of key genes and biological pathways in SCLC.

In this study, gene expression data obtained from GEO databases were integrated to conduct data mining and analysis of SCLC. Then, a series of co-differentially expressed genes have been screened in SCLC. A series of analysis were carried out based on these genes, including analysis of functional enrichment, protein-protein interaction network and human samples validation. We identified numbers of hub genes analyzed the interaction between genes and drugs. Our research may offer more insight into the molecular mechanisms or study of available drugs for this epidemic and destructive disease. The workflow for bioinformatics strategy of SCLC was illustrated in Fig 1.

## Materials and methods

### SCLC gene expression data from GEO data repository

As a publicly genomics database, Gene Expression Omnibus (GEO) of NCBI (https://www.ncbi.nlm.nih.gov/) collects submitted high-throughput gene expression data and can be used for retrieving all datasets involving studies of SCLC. For our following analysis, it was considered reasonable for studies that met the following criteria: (1) Studies of human SCLC and corresponding adjacent or normal lung tissues. (2) There are detailed information of research technology and platform. (3) All studies have been published in English. Based on these criteria, three gene expression microarray datasets for SCLC, including GSE40275, GSE99316, and GSE60052, were taken from GEO. Details of these microarray studies were shown in Table 1.

**Table 1:** Details of associated microarray datasets from GEO database in SCLC studies.

GSE	Reference PMID	Platform	Sample		Tumor type	Country	Lastest update
			Normal lung	Tumor			
GSE40275	None	GPL15974 Human Exon 1.0 ST Array [CDF: Brainarray Version	43	15	SCLC	Austria	2012
		9.0.1, HsEx10stv2_Hs_REFSEQ]					
GSE99316	23714854	GPL570 [HG-U133_Plus_2]	1	23	SCLC	Japan	2019
		Affymetrix Human Genome					
		U133 Plus 2.0 Array					
GSE60052	27093186	GPL11154 Illumina HiSeq 2000	7	79	SCLC	USA	2019
		(Homo sapiens)					

SCLC, small cell lung cancer.

### Data preprocessing and DEGs screening

The DEGs between SCLC and normal lung samples from GEO datasets, GSE40275 and GSE99316, were screened by using GEO2R (http://www.ncbi.nlm.nih.gov/geo/geo2r). GEO2R is an online analysis tool for comparing two or more groups of data in the GEO series to identify DEGs under the same detection method. The matrix data (.TSV, from supplementary files) of GSE60052 were normalized and log_2_ transformed by limma package of R [13]. The application of the adjusted *P*-value (adj. *P*) can balance the detection of statistically significant genes and the restriction of false positive. Probe sets without gene symbols were removed, and genes with multiple probe sets were averaged. Next, |log2FC (fold change) |>1 and adj. *P*-value<0.05 was considered to be statistically significant. To visualize the identified DEGs, SangerBox (soft.sangerbox.com) and FunRich [14] were used to make volcano plots and Venn diagrams, respectively.

### Circular visualization of the DEGs

Circos (http://circos.ca/) was applied to display our data for a better understanding of DEGs, including their gene symbols and locations on chromosomes [15].

### Functional enrichment analysis of DEGs

Funrich is a publicly available software for Functional annotation and pathway enrichment analysis of genes or proteins. In this study, Funrich was used to analyze the functional enrichment of up-regulated and down-regulated DEGs, including molecular function, biological process, cell composition and biological pathway. The results of the biological pathway analysis were visualized by OmicShare tools (http://www.omicshare.com/tools), a free online platform for data analysis.

### Construction and analysis of protein-protein interaction (PPI) network

For the better view of the relationship among these DEGs, the PPI network was constructed via using The Search Tool for the Retrieval of Interacting Genes (STRING) database (version 11.0; http://string-db.org/) [16]. In the present study, Cytoscape software (version 3.6.1) was used to establish and visualize PPI networks [17]. As one of the characteristics of PPI network, the network module contained specific biological significance. The salient modules in this PPI network were explored by The Cytoscape plug-in Molecular Complex Detection (MCODE) [18]. The thresholds were set as follows: Degree cutoff = 2, Node Score Cutoff = 0.2, and K-Core = 2. Next, the pathway enrichment analysis of the DEGs in different modules was performed by FunRich and visualized by the OmicShare tools.

### Screening of hub genes

The hub genes in the PPI network were screened by cytohubba, a plug-in of Cytoscape software (version 3.6.1). The genes with degree score ≥75 were considered as hub genes.

### Verification of hub genes expression levels

At first, the expression of these hub genes was validated in Oncomine database. As one of the world’s largest tumor gene microarray database and integrated data analysis platform, Oncoline (oncomine.org) is designed to excavate cancer genetic information. The database hitherto has collected data from 729 gene expression data sets and over 90,000 cancer and normal tissue samples. It can be used to uncover the differential expression of a single gene in SCLC tissue and its related normal tissues [19]. To figure out the expression levels of hub genes in SCLC, SCLC gene expression data from the study of Garber et al [20] and Bhattacharjee et al [21] in the Oncomine database were investigated and visualized by GraphPad Prism. Thresholds for the data type was restricted to mRNA. Then, the expression levels of hub genes were further verified by quantitative real-time PCR (RT-qPCR) through tissue samples of SCLC patients and paired adjacent non-cancerous ones.

### Construction and analysis of hub gene-drug interaction network

The hub gene-drug interaction networks were constructed by Comparative Toxicogenomics Database (CTD) [22], a platform for analysis chemotherapeutic drugs which could inhibit or induce the mRNA or protein expression of hub genes. The hub gene-drug interaction was investigated in CTD database and visualized by the OmicShare tools.

### Human SCLC samples

All 7 SCLC tissues and 7 paired adjacent non-cancerous tissue samples of PPFE (Formalin-fixed and paraffin embedded) were collected from patients who had been diagnosed as SCLC from May 2019 to May 2020 at Taihe Hospital of Hubei University of Medicine, China. The PPFE samples were stored at room temperature until total RNA was extracted. All SCLC patients were diagnosed and graded according to the pathological characteristics in the Department of Pathology, Taihe Hospital. All human samples were obtained by informed consent (IFC) from patients, and this study was supported and approved by by the Ethics Committee of Taihe Hospital.

### RNA isolation and reverse transcription PCR

Quantitative real-time PCR (RT-qPCR) were performed according to methods published previously [23, 24]. Total RNA was extracted from the PPFE samples by using RNeasy FFPE Kit (cat. no. 73504, QIAGEN, Germany). Total RNA of 1 μg was reversely transcribed in a 20 μl reaction using RevertAid First Strand cDNA Synthesis Kit (cat. no. #K1622, Thermo Scientific, USA) according to the manufacturer’s protocol. The reaction products were then diluted with 40 μl RNase-free water. The real-time PCR reaction was composed of 2 μl cDNA, 10 μl of PowerUp^™^ SYBR^™^ Green Master Mix (cat. no. A25741, Thermo Scientific, USA) and 0.5 μl of forward and reverse primers (0.5 μM). RT-qPCR was conducted in an ABI Prism 7500 analyzer (Applied Biosystems, USA) for 40 cycles (95°C for 15 sec, 58°C for 15 sec, 72°C for 30 sec) after an initial 120s denaturation at 95°C. HPRT1 was endogenous reference gene. All reactions were run in triplicate. The relative RNA levels of SCLC samples were calculated by using the 2^−ΔΔCt^ method. All primers of the hub genes and HPRT1 were synthesized by Sangon Biotech (Shanghai, China), and the information of their sequences were listed in Table 2.

**Table 2:** RT-PCR primers of 8 most significant hub genes and reference gene, HPRT1.

Gene name	Full name	Primer sequence(5'→3')	Tm(°C)	Product(bp)
***CDC20***	cell division cycle 20, aliases: CDC20A	Forward: AGTTCGCGTTCGAGAGT	55.7	195
		Reverse: GAACCTTGGAACTGGAT	50	
***BUB1***	budding uninhibited by benzimidazoles-1, aliases: BUB1A	Forward: TATAGCAGGCTGATTGGGCT	58.5	107
		Reverse: TGGCTTAAACAGGTCAGTGT	57	
***TOP2A***	Topoisomerase II alpha	Forward: GTGTCACCATTGCAGCCTGT	61	152
		Reverse: GAACCAATGTAGGTGTCTGG	56	
***UBE2C***	ubiquitin conjugating enzyme E2 C	Forward: CTCATGGTATATGAAGACCT	51	132
		Reverse: GCATATGTTACCCTGGGTGT	57	
***BUB1B***	BUB1 mitotic checkpoint serine/threonine kinase B	Forward: TGCTCTGAGTGAAGCCATGT	59	99
		Reverse: TGAAGCGTGGACATGATCCG	60	
***RRM2***	ribonucleotide reductase regulatory subunit M2	Forward: GGGAATCCCTGAAACCCGAG	60	70
		Reverse: CCATCGCTTGCTGCAAAGAA	59.7	
***CCNA2***	cyclin A2	Forward: GGATGGTAGTTTTGAGTCACCAC	59	202
		Reverse: CACGAGGATAGCTCTCATACTGT	59	
***MAD2L1***	mitotic arrest deficient 2 like 1	Forward: ACGGTGACATTTCTGCCACT	60	105
		Reverse: TGGTCCCGACTCTTCCCATT	60.5	
***HPRT1***	hypoxanthine phosphoribosyl-transferase 1	Forward: GGACTAATTATGGACAGGACTG	55	195
		Reverse: GCTCTTCAGTCTGATAAAATCTAC	55	

### Statistical analysis

Statistical analysis was performed through GraphPad Prism (version 8.2.1, San Diego, CA) software. Student’s t-tests were used for the comparison of two sample groups. Differences were considered as statistically significant when *P* < 0.05 (*P < 0.05, ** *P* < 0.01, *** *P* < 0.001).

## Results

### 1 Identification of DEGs in SCLC

**Fig 2:**
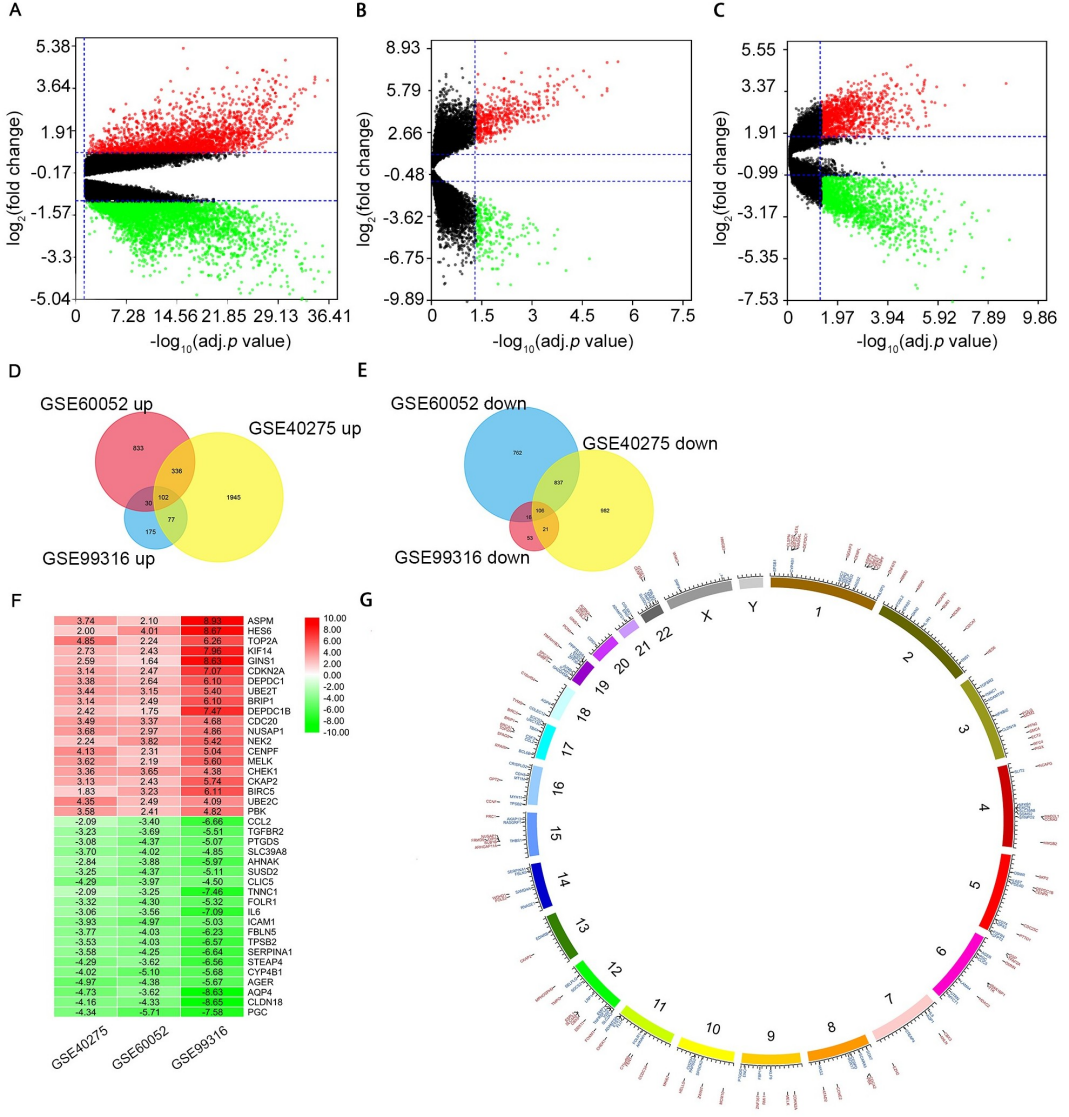
Identification of differentially expressed genes (DEGs) between SCLC and normal tissues. Volcano plots displayed the number of DEGs verified from the three GEO datasets. (A) volcano plots of GSE40275, (B) volcano plots of GSE99316, (C) volcano plots of GSE60052. Green denotes a lower expression level, red denotes a higher expression level and black denotes that the gene has no different according to the cut-off criteria. Venn diagram demonstrates the overlapping DEGs, including (D) 102 upregulated and (E) 108 downregulated genes among the three datasets. (F) Heat map of top20 DEGs for upregulated and downregulated genes. Green denotes a lower expression level, red denotes a higher expression level and black denotes that the gene has no different according to the cut-off criteria. Each column denotes a dataset and each row denotes a gene. The number in each rectangle denotes a normalized gene expression level. (G) Chromosome mapping of DEGs. Blue denotes down-regulated DGEs, and red denotes up-regulated DGEs.

117 SCLC samples and 51 normal lung samples were involved in this study (Table 1 and S1 Table). There were 3337 DEGs (1752 upregulated and 1585 down-regulated) in GSE40275, 510 DEGs (326 up-regulated and 184 down-regulated) in GSE99316-GPL570, and 2304 DEGs (953 up-regulated and 1351 down-regulated) in GSE60052 which were identified between SCLC tissues and normal lung tissues as shown in volcano plots (Fig 2A–2C). The Venn diagram analysis of these DEGs mapped that 208 DEGs, including 102 up-regulated genes and 106 down-regulated genes, were consistently found in the three data sets (Fig 2D–2E and S2 Table). All 208 DEGs are listed in Table 3. As shown in Fig 2F, we screened top 20 differentially expressed up-regulated and down-regulated genes respectively by the cut-off criteria.

**Table 3:** Two hundred and eight differentially expressed genes (DEGs) were identified and confirmed from three GEO datasets, including 102 up-regulated genes and 108 down-regulated genes in SCLC tissues, compared to normal lung tissues.

Regulation	Number	DEGs (Gene symbol)
**Up-regulated**	102	*TOP2A*,*UBE2C*,*CENPF*,*PRC1*,*ASPM*,*NUSAP1*,*EZH2*,*CCNE2*,*HELLS*,*MELK*,*PBK*,*CDC20*,*TYMS*,*ARHGAP11A*,*UBE2T*,*DEPDC1*,*KIF2C*,*CHEK1*,*KIAA0101*,*MKI67*,*BRIP1*,*CDKN2A*,*CKAP2*,*HMGB3*,*ANLN*,*NCAPG*,*PTTG1*,*RFC4*,*MSH2*,*BUB1B*,*CENPK*,*ATAD2*,*TTK*,*PFN2*,*BUB1*,*CCNA2*,*RRM2*,*AURKA*,*ECT2*,*KIF14*,*WDHD1*,*HMGB2*,*STIL*,*PCNA*,*KIAA1524*,*MPHOSPH9*,*GINS1*,*UHRF1*,*ZWINT*,*SPAG5*,*DEPDC1B*,*GMNN*,*RMI1*,*MPO*,*POLE2*,*DSP*,*NEK2*,*MCM6*,*MCM2*,*CLSPN*,*SMC4*,*WNK3*,*CBX3*,*FOXM1*,*POLQ*,*HES6*,*CENPL*,*BRCA1*,*CDCA7*,*CDC25C*,*CDCA2*,*HDAC2*,*BIRC5*,*MAD2L1*,*C11orf80*,*RAD54L*,*IQGAP3*,*SKP2*,*FEN1*,*CBX5*,*PIGX*,*MYBL2*,*NCAPH*,*CCDC34*,*PAFAH1B3*,*GTSE1*,*ESPL1*,*GPT2*,*SLC4A8*,*TFAP2A*,*ZNF670*,*CCNF*,*OIP5*,*MCM10*,*C18orf54*,*CENPM*,*RPAIN*,*FRMD5*,*IRAK1BP1*,*ZNF367*,*DDX11*,*SPC24*
**Down-regulated**	106	*NFKB1*,*SOX17*,*DDR2*,*NLRP3*,*SPSB1*,*WISP2*,*GFPT2*,*MAFF*,*PDE4D*,*COL6A1*,*IL6ST*,*BCL6B*,*AKAP13*,*SOX7*,*ETS1*,*SLC2A3*,*CRISPLD2*,*CSF3*,*SOCS2*,*CH25H*,*UNC13D*,*SAMD4A*,*TNFRSF1A*,*SCARA5*,*ADAMTS8*,*KLF9*,*NFKBIZ*,*RASGRF1*,*FLI1*,*AHNAK*,*FBLN1*,*SRPX*,*SYNPO2*,*EHD2*,*THBS1*,*FOSL2*,*LAMA4*,*DAMTS9*,*GUCY1A3*,*LTBP4*,*FGFR4*,*CEBPD*,*TNNC1*,*CCL2*,*LRP1*,*TBX4*,*SELPLG*,*ERG*,*SLIT2*,*RNASE1*,*JUNB*,*HAS2*,*TMEM2*,*MYCT1*,*PPP1R15A*,*GADD45B*,*TIMP3*,*SELP*,*OSMR*,*RGS2*,*CD74*,*UTRN*,*TNS1*,*PTRF*,*COLEC12*,*EMCN*,*ENG*,*MT1M*,*MYH11*,*EMP1*,*SPOCK2*,*CD93*,*MUC1*,*SOCS3*,*IL1R1*,*FHL1*,*CDH5*,*FBP1*,*VWF*,*IL6*,*PTGDS*,*ZFP36*,*ADAMTS1*,*TGFBR2*,*SUSD2*,*PAPSS2*,*SGMS2*,*FOLR1*,*EDNRB*,*TPSB2*,*GKN2*,*SERPINA1*,*AQP1*,*SLC39A8*,*FBLN5*,*FMO2*,*GPX3*,*ICAM1*,*CYP4B1*,*EPAS1*,*CLDN18*,*CLIC5*,*STEAP4*,*PGC*,*AQP4*,*AGER*

Chromosome mapping of DEGs presented gene distribution on chromosomes, with chromosomes 1 containing the most dysregulated genes in SCLC (Fig 2G). Interestingly, four genes showed dysregulation on the X chromosome in SCLC (*FHL1*, *SRPX*, *HMGB3* and *WNK3*), while no genes on the Y chromosome was affected.

### 2 Functional annotation and pathway enrichment analysis

Funrich, as a tool for the analysis of genes and proteins, was used for GO functional annotation and biological pathway enrichment analysis of DEGs.

**Fig 3:**
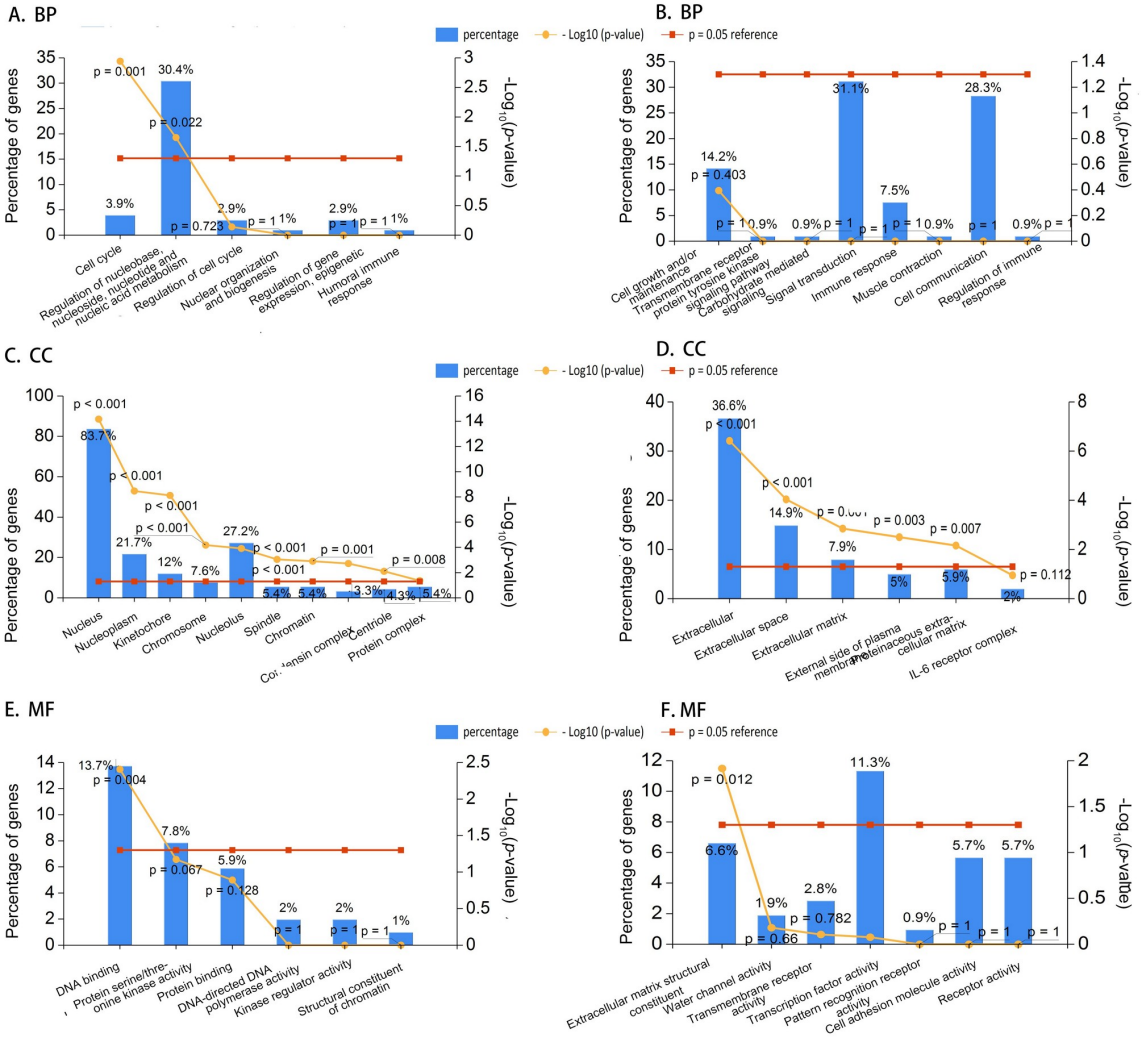
Functional annotation and pathway enrichment analysis for DGEs. (A) BP enrichment of up-regulated DEGs; (B) BP enrichment of down-regulated DEGs; (C) CC enrichment of up-regulated DEGs; (D) CC enrichment of down-regulated DEGs; (E) MF enrichment of up-regulated DEGs; (F) MF enrichment of down-regulated DEGs. X axis denotes detailed appellations of functional annotation and pathway enrichment; Y axis denotes percentage of genes or–log10(*P*-value). Red line denotes *P* = 0.05, the reference value for cut-off criteria of statistical analysis; Yellow line denotes–log10(*P*-value).

There are three categories, including biological process (BP), cellular component (CC) and molecular function (MF), involved in GO functional annotation. In Fig 3, the top 10 enriched GO projects were displayed. Analysis of GO BP suggested that up-regulated DEGs were significantly enriched in cell cycle, regulation of nucleobase, nucleoside, nucleotide and nucleic acid metabolism (Fig 3A). For analysis of CC, the genes were significantly enriched in nucleus, nucleoplasm, kinetochore, and chromosome (Fig 3C). The analysis of MF for these genes mainly included DNA binding (Fig 3E).

Analysis of GO BP indicated that down-regulated DEGs were most but not significantly enriched in cell growth and/or maintenance, and transmembrane receptor protein tyrosine kinase signaling pathway (Fig 3B). For analysis of CC, the genes were manifestly enriched in extracellular, extracellular space, and extracellular matrix (Fig 3D). Finally, analysis of MF of these genes showed that they were apparently enriched in extracellular matrix structural constituents (Fig 3F).

**Fig 4:**
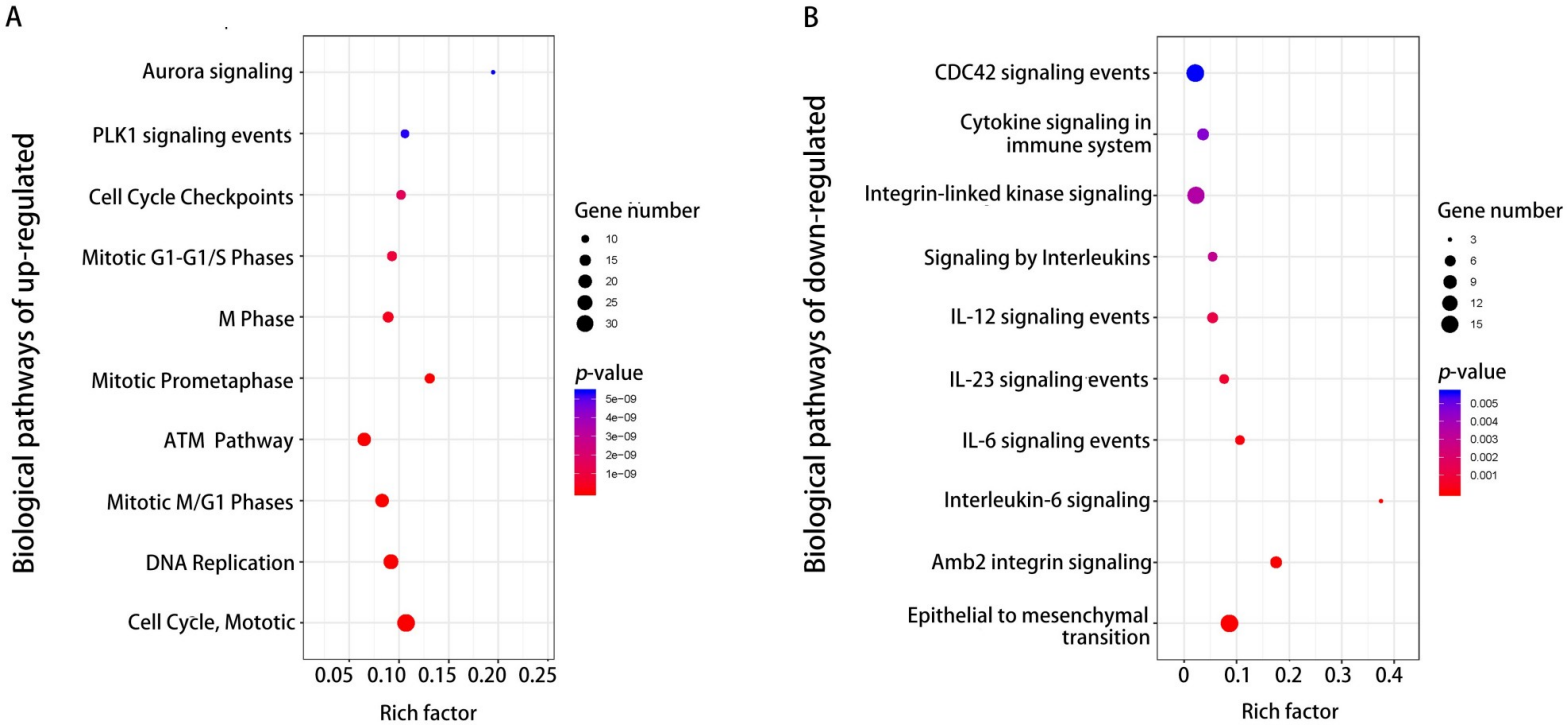
Enriched biological pathways of up-regulated DEGs (A) and down-regulated DEGs (B).

Furthermore, pathway enrichment analysis was carried out for up-regulation and down-regulation DEGs. Candidate genes of up-regulated DEGs were mainly enriched in mitotic cell cycle/ M-M/G1 phases, DNA replication, and ATM pathway (Fig 4A and Table 4). Moreover, a critical gene *CDC20* was particularly enriched in cell cycle and DNA replication in pathway enrichment analysis for up-regulated genes (Table 4 and S3 Table). The notably enriched pathways for down-regulated DEGs were epithelial-to-mesenchymal transition, amb2 integrin signaling, and interleukin-6 (*IL-6*) signaling pathway (Fig 4B). However, Interleukin-6 (*IL-6*), a crucial gene associated with inflammation was significantly enriched in amb2 Integrin signaling, Interleukin-mediated signaling, and cytokine signaling in the immune system in pathway enrichment analysis for down-regulated genes (Table 4 and S3 Table).

**Table 4:** The top 10 enriched biological pathways of up-regulated and down-regulated DEGs.

Term of up-regulated DEGs	Overlap	*P*-value	Term of down-regulated DEGs	Overlap	*P*-value
Cell Cycle, Mitotic	34/317	5.58E-28	Epithelial-to-mesenchymal transition	16/185	6.33E-11
DNA Replication	24/261	1.36E-17	amb2 Integrin signaling	7/40	1.77E-07
Mitotic M-M/G1 phases	20/242	1E-13	Interleukin-6 signaling	3/8	6.51E-05
ATM pathway	20/307	8.88E-12	IL6-mediated signaling events	5/47	0.000137
Mitotic Prometaphase	13/99	1.1E-11	IL23-mediated signaling events	5/66	0.000683
M Phase	14/158	3.62E-10	IL12-mediated signaling events	6/111	0.001187
Mitotic G1-G1/S phases	13/140	9.35E-10	Signaling by Interleukins	5/92	0.003028
Cell Cycle Checkpoints	12/118	1.56E-09	Integrin-linked kinase signaling	15/154	0.00356
PLK1 signaling events	11/104	5.3E-09	Cytokine Signaling in Immune system	7/193	0.004573
Aurora B signaling	8/41	5.38E-09	CDC42 signaling events	16/755	0.005622

### 3 Analysis of PPI network and modules

**Fig 5:**
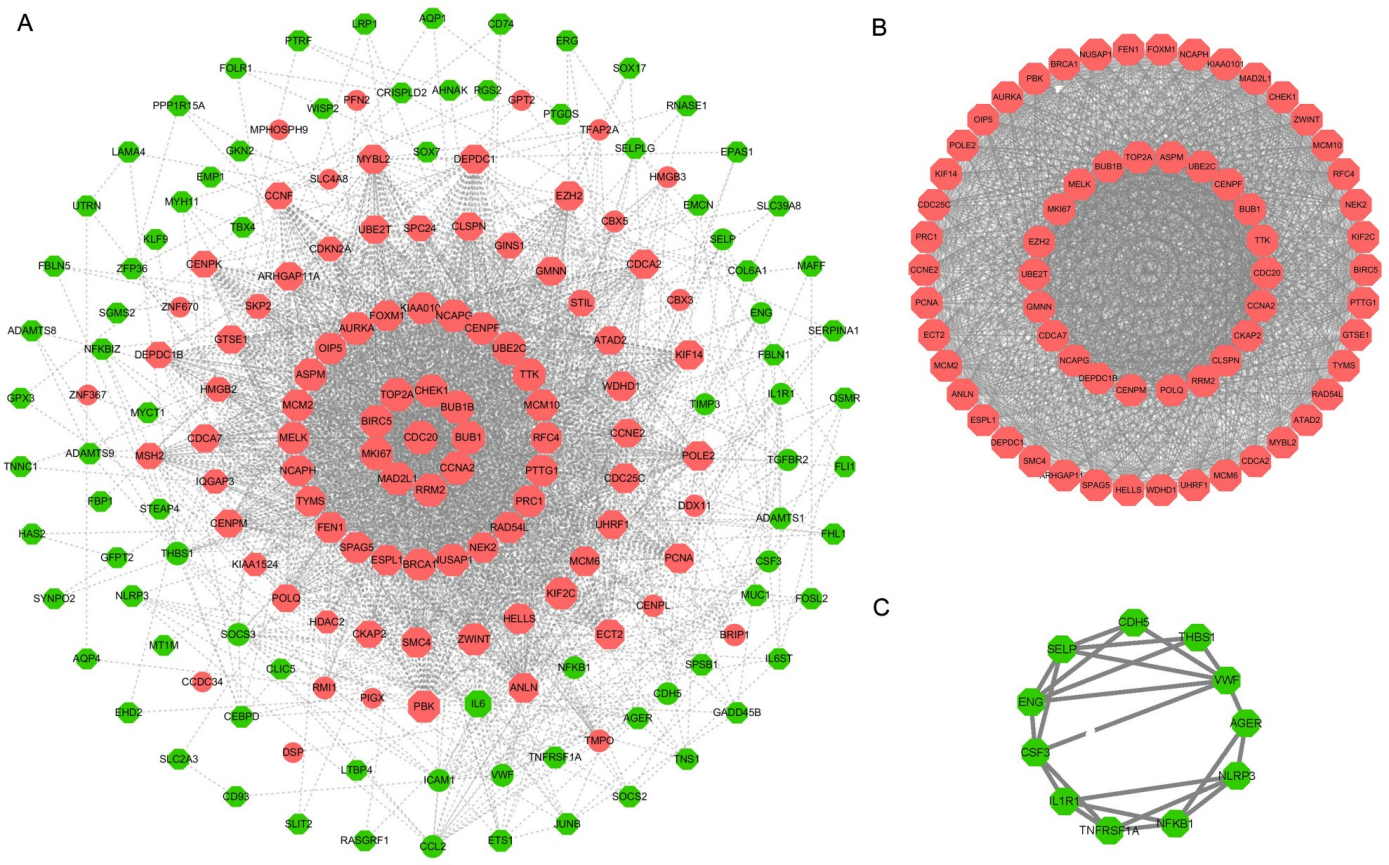
Construction and analysis of PPI network. (A) PPI network constructed by the DEGs of three GEO datasets. The significant modules, module1(B) and module2(C), identified from the PPI network by MCODE method. The red nodes denote upregulated DEGs, and the green nodes denote downregulated DEGs.

As results, the PPI network showed that a total of 178 nodes and 2466 protein pairs were acquired with a score > 0.4. The main nodes in this network were the up-regulated DEGs (Fig 5A and S4 Table). Furthermore, two modules (module 1 and module 2) with score >4 were detected by MCODE (Fig 5B–5C). All nodes in module 1 with an MCODE score of 56.094 (65 nodes, 1795edges) were up-regulated DEGs, while all nodes in modules 2 with an MCODE score of 4.60 (11nodes, 23edges) were down-regulated DEGs in SCLC samples.

**Fig 6:**
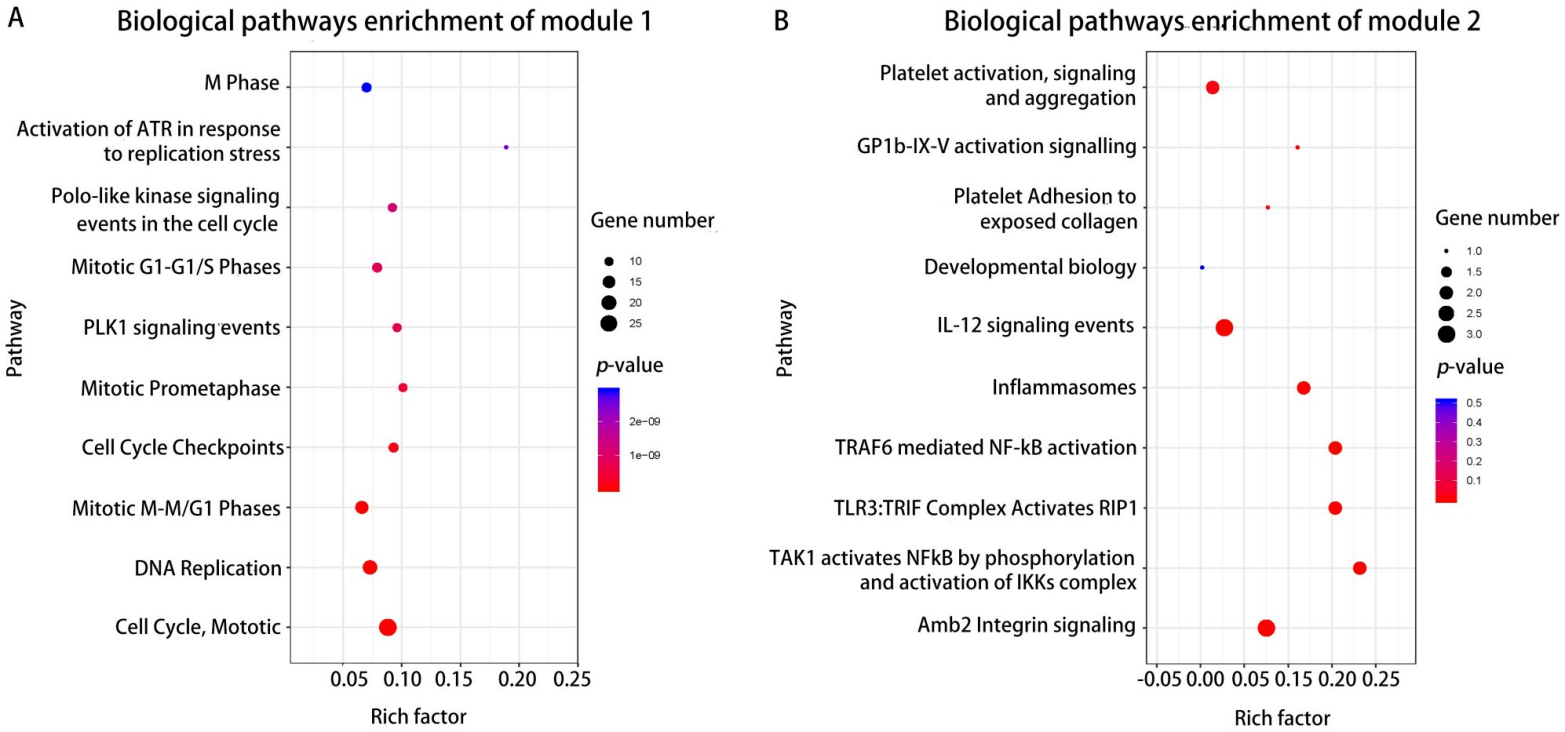
Biological pathway enrichment analysis of two modules. The top 10 biological pathways enrichment analysis of DEGs in module 1(A) and module 2(B) by FunRich, respectively, P < 0.05. X axis denotes rich factor, namely the enrichment levels; Y axis denotes the top 10 significantly enriched pathways of two modules. The color of the dot indicates the different *P*-value, and the size of the dot denotes the number of candidate genes enriched in the corresponding pathway.

Furthermore, the biological pathways enrichment analysis of two modules were shown in Fig 6 and S5 Table. The mitotic cell cycle pathway was identified as the most significant pathway in module 1 (Fig 6A), and amb2 Integrin signaling was the most significant pathway in module 2 (Fig 6B).

### 4 Hub genes screening

For seeking hub genes in the PPI networks, all these node pairs were identified by cytohubba. As shown in Table 5, the genes, *CDC20*, *BUB1*, *TOP2A*, *RRM2*, *CCNA2*, *UBE2C*, *MAD2L1*, and *BUB1B*, were hub genes with higher node degrees, and they were all up-regulated genes in module 1.

**Table 5:** Hub genes with high node degrees.

Gene symbol	Degree score	Type	MCODE cluster
*CDC20*	78	Up-regulated	Cluster 1
*BUB1*	77	Up-regulated	Cluster 1
*TOP2A*	77	Up-regulated	Cluster 1
*RRM2*	76	Up-regulated	Cluster 1
*CCNA2*	76	Up-regulated	Cluster 1
*UBE2C*	76	Up-regulated	Cluster 1
*MAD2L1*	75	Up-regulated	Cluster 1
*BUB1B*	75	Up-regulated	Cluster 1

### 5 Verification of hub gene mRNA expression levels

**Fig 7:**
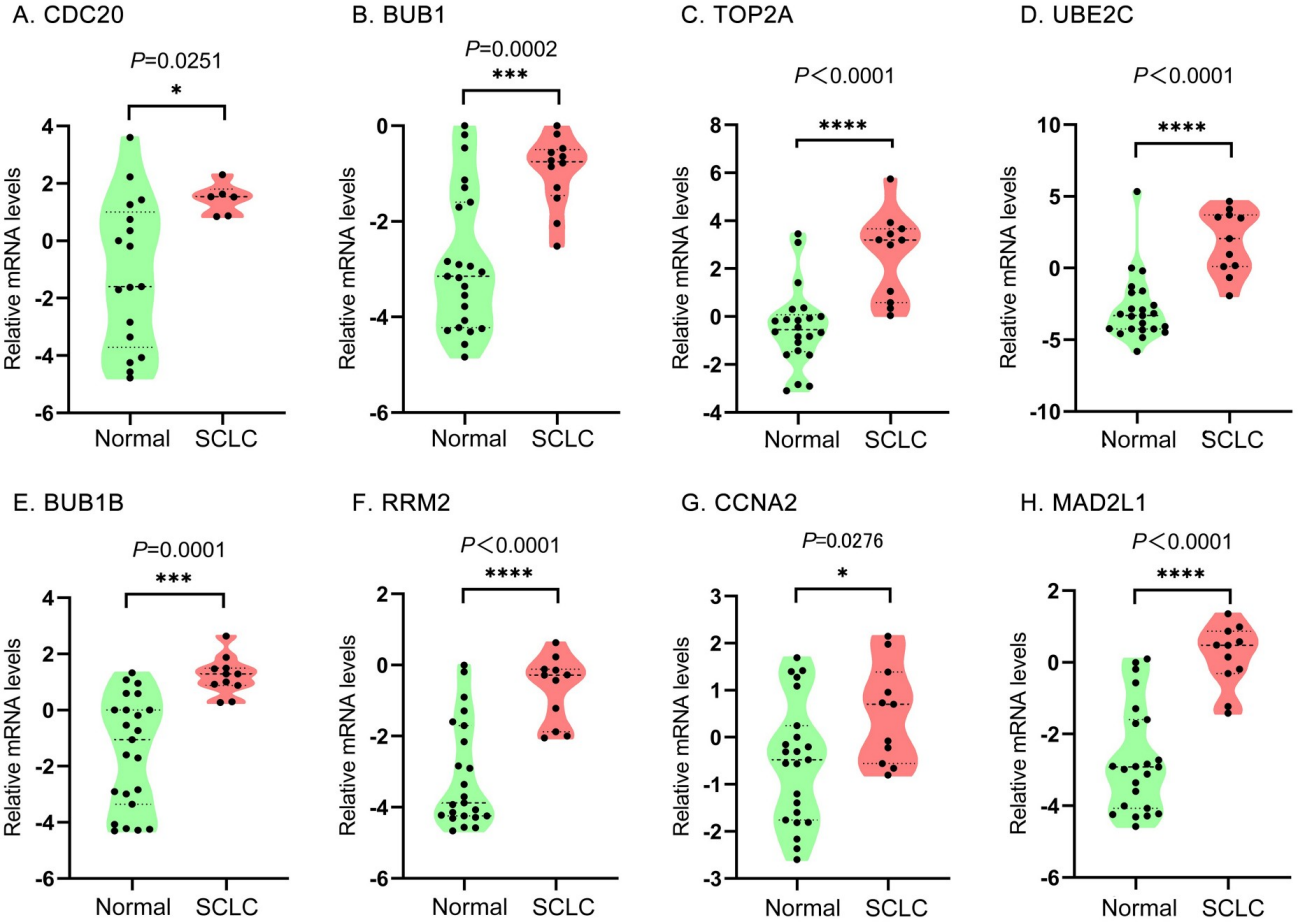
Analysis of expression of hub genes in the Oncomine database. Relative mRNA expression levels of (A) CDC20, (B) BUB1, (C) TOP2A, (D) UBE2C, (E) BUB1B, (F) RRM2, (G) CCNA2, and (H) MAD2L1 in normal lung and SCLC samples. The expression level data were standardized by log_2_ conversion and median centered. Data are presented as violin plot with minimum (from bottom to top), 25th percentile, median, 75th percentile, and maximum. The black dots denote the sample size. Data were analyzed using paired student’ s t-test. Differences were considered as statistically significant when *P* < 0.05 (*P < 0.05, ** *P* < 0.01, *** *P* < 0.001).

**Fig 8:**
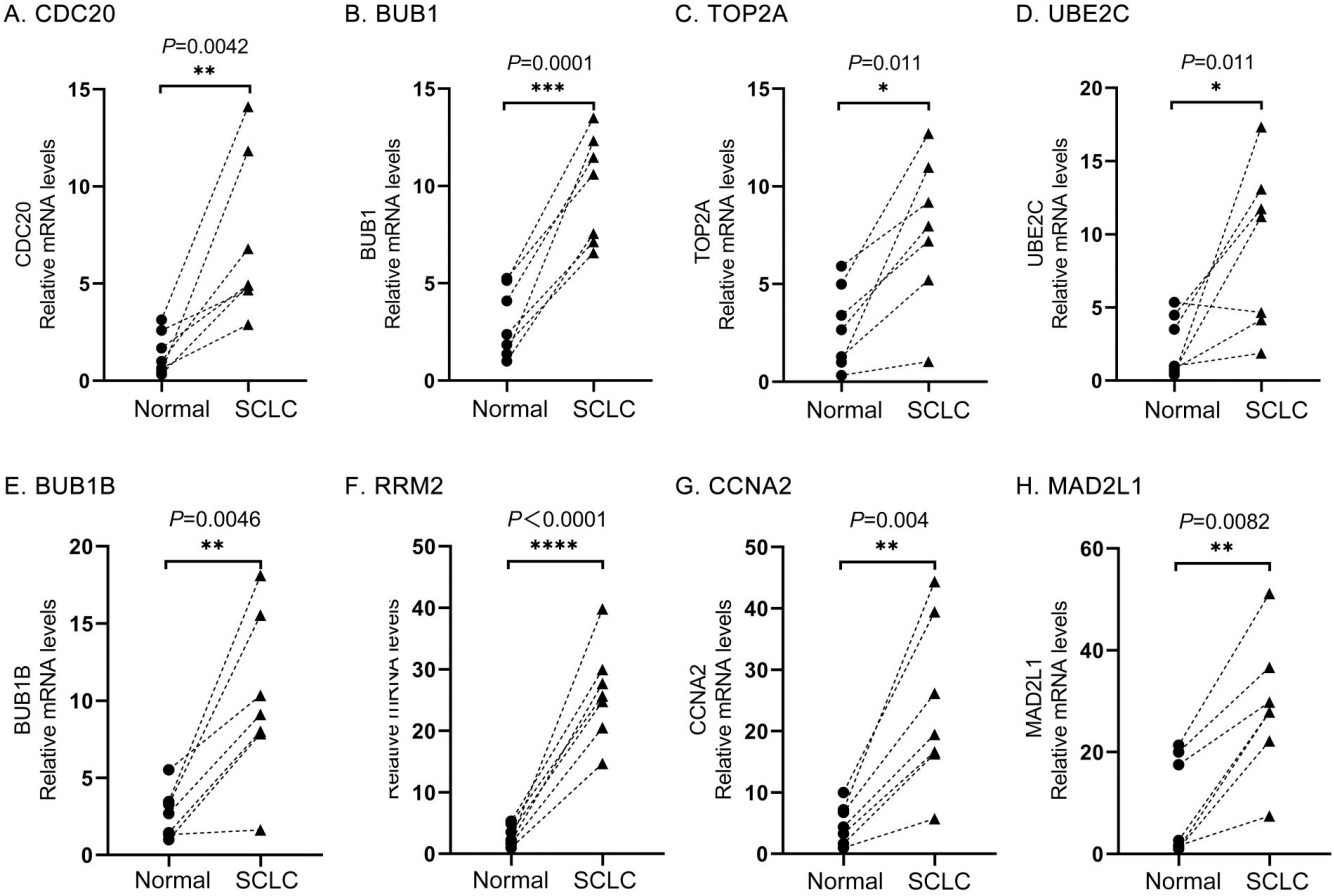
The relative expression of eight hub genes in human SCLC samples (n = 7) and paired adjacent non-cancerous tissue samples (n = 7) were detected using RT-qPCR. (A) CDC20, (B) BUB1, (C) TOP2A, (D) UBE2C, (E) BUB1B, (F) RRM2, (G) CCNA2, and (H) MAD2L1. HPRT1 was internal reference gene.analyze.

Firstly, the mRNA expression levels of *CDC20*, *BUB1*, *TOP2A*, *RRM2*, *CCNA2*, *UBE2C*, *MAD2L1*, and *BUB1B* were significantly increased in SCLC samples compared with normal lung samples based on the Oncomine database, which was consistent with the above bioinformatics investigation (Fig 7). Secondly, further study was conducted to verify the expression levels of these hub genes by RT-qPCR through tissue samples of SCLC patients and paired adjacent non-cancerous ones. The mRNA levels of eight hub genes in SCLC tissues were significantly overexpressed compared to those in paired adjacent ones. (Fig 8).

### 6 Analysis of hub gene-drug interaction network

**Fig 9:**
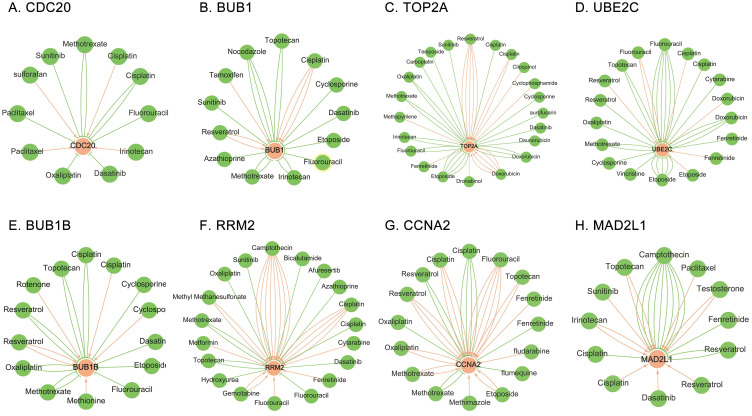
Construction of gene-drug interaction network among eight hub genes and corresponding available chemotherapeutic drugs. The available chemotherapeutic drugs could induce or reduce the expression level of these hub genes, including (A) CDC20, (B) BUB1, (C) TOP2A, (D) UBE2C, (E) BUB1B, (F) RRM2, (G) CCNA2, and (H) MAD2L1, in mRNA or protein. The red arrow denotes that chemotherapy drugs promote the expression of hub gene; the green arrow indicates that chemotherapy drugs inhibit the expression of hub gene. In this network, the number of arrows between drugs and genes represents the number of supports in previous literature.

CTD was used to study the interaction between hub genes and available therapeutic drugs of cancer. As results, multiple drugs could alter the expression of these eight hub genes, including *CDC20*, *BUB1*, *TOP2A*, *RRM2*, *CCNA2*, *UBE2C*, *MAD2L1*, and *BUB1B*. For example, Sunitinib, Methotrexate and Fluorouracil could inhibit the expression of *CDC20* while Irinotecan could promote the expression of *CDC20* (Fig 9A).

## Discussion

Due to the insufficiency in effective targeted therapy options, SCLC is considered a “neglected sibling” compared to NSCLC. The mutations of epidermal growth factor receptor (*EGFR*) kinase, the fusion of the anaplastic lymphoma kinase (*ALK*), and ROS Proto-Oncogene 1 Receptor Tyrosine Kinase (*ROS1*), result in an immense improvement in the remedy of sufferers with NSCLC [25–28]. However, these genetic variations are not common in SCLC, and targeted drugs for NSCLC are not effective for SCLC [29]. Therefore, novel biomarkers with high efficiency, high sensitivity and high specificity are urgently needed for diagnosis and prognosis of SCLC.

Compared with single array analysis, multiple arrays integration is considered as a better method to enhance detection capabilities and improve the reliability of results [30]. In this study, we analyzed three SCLC data sets from GEO to gain insight into gene expression patterns on a genome-wide scale. Then, 208 DEGs (102 up-regulated and 108 down-regulated) and eight hub genes have been identified and used for further analysis. Chromosome mapping of 208 DEGs displayed that chromosome 1 contained the most dysregulated genes in SCLC. Previously studies affirmed that early-stage development of lung cancer was associated with X chromosome inactivation in females. The inactivation test of X-chromosome could be used to screen women who are prone to malignant tumors, including lung cancer [31]. Our findings suggested that the abnormal expression of *FHL1*, *SRPX*, *HMGB3* and *WNK3* on the X chromosome may be related to SCLC in females [32]. However, there is no differential expression gene in Y chromosome in our present study.

A PPI network was constructed for the purpose of predicting the protein functional association of 208 identified DEGs. As a result, up-regulated genes were predominantly enriched in mitotic cell cycle, DNA replication, mitotic M-M/G1 phases, and ATM pathway in SCLC. Meanwhile, down-regulated genes were mainly enriched in epithelial-to-mesenchymal transition, amb2 Integrin signaling, and *IL-6* signaling pathway in SCLC. Among the top 10 biological pathways enrichment analysis for down-regulated genes, 9 pathways were closely related to IL-6 and immune system. (Table 4 and S3 Table) It indicated that the immune level of SCLC was relatively low, and application of IL-6 inhibitors, immunotherapy or activation of immunity might be the potential strategies for the treatment of SCLC.

In the current study, the eight hub genes selected by cytohubba were overexpressed in SCLC tissues compared to paired adjacent non-cancerous tissues. Lastly, seven of those hub genes, namely *CDC20*, *TOP2A*, *BUBI*, *BUBIB*, *UBE2C*, *CCNA2* and *MAD2L1* (all *P*-values <0.05), were closely related to the cell cycle pathway. Additionally, the mRNA expression of hub genes were searched for by mining the Oncomine database and human samples, which further validated the bioinformatics analysis. Although previous studies have authenticated that most of these disordered hub genes were closely related to the diagnosis, treatment and prognosis of various diseases, the precise functions and molecular mechanism of them in the occurrence and development of SCLC have not yet been clearly illuminated.

As an evolutionarily conservative process, cell cycle plays an imperative role in cell growth and differentiation. Dysregulations of cell cycle are considered as a hallmark of human cancer [33]. In the treatment of cancer, many strategies for the cell cycle have been implemented. Increasingly studies have revealed that several genes related to cell cycle such as *CDC20*, *TOP2A*, *BUBI*, *BUBIB*, and *UBE2C*, are related to the occurrence and development of cancer, which were also identified in the present study.

Cell division cycle protein 20 (*CDC20*), locating in chromosome 1, is a promoter for the anaphase-promoting complex (APC). Early research has demonstrated that *CDC20* is highly expressed in multiple tumors, and is related to the poor prognosis of patients with gastric cancer, liver cancer, bladder cancer, colon cancer [34], and NSCLC [35]. Subsequent studies have indicated that the inhibition of CDC20 expression could reduce the colony formation rate of lung cancer cells in NSCLC and SCLC [36]. Therefore, *CDC20* may be viewed as a novel or latent target for the amelioration of SCLC.

Topoisomerase II alpha (*TOP2A*) gene, encoding a 170 kDa nuclear enzyme that regulates the DNA topological state during the process of DNA transcription and replication. It catalyzes the fracture and reconnection of double stranded DNA, thus changing the topological structure of DNA [37]. Numerous studies indicated that *TOP2A* is highly expressed in a variety of malignant tumors, such as colorectal cancer [38], meningioma [39], breast cancer [40], adrenocortical carcinoma [41], NSCLC [42] and SCLC [43, 44]. Topoisomerase II Inhibitors have been illustrated as active agents in SCLC cell lines [45, 46], and levels of *TOP2A* are important determinants of drug response in SCLC [47]. In our current study, *TOP2A* was overexpressed in SCLC and mostly enriched in mitotic cell cycle pathway. Consequently, the results of our study are in concert with those of previous studies, suggesting that *TOP2A* may be a direct or indirect factor in the occurrence and deterioration of SCLC

The multidomain protein kinases *BUB1* (aliases: *BUB1A*) and *BUB1B* (aliases: *BUBR1*) are key elements of the mitotic checkpoint for spindle assembly [48]. *BUB1* plays an important role in chromosome assembly and kinetochore localization in cells [49–51]. While *BUB1B* is associated with stabilizing centromere-microtubule junction and chromosome alignment [52]. Upregulation of *BUB1B* can prevent aneuploidy and cancer and prolong healthy lifetime [53]. Abnormal expression of *BUB1* and *BUB1B* were resulted in the prognosis of patients with brain tumor [54], glioblastoma [55], colorectal cancer [56], and NSCLC [57], and resulted in the impairment of mitotic checkpoint function. Therefore, future studies on *BUB1* and *BUB1B* combining genetic approaches may provide an effective strategy for clinical anti-tumor treatment and could be the focus of SCLC research in the future.

As reported, ubiquitin-conjugating enzyme E2C (UBE2C), belonging to the E2 ubiquitin-conjugating enzyme family, plays crucial roles in a variety of malignancies, namely breast cancer [58], colorectal cancer [59], melanoma [60], and hepatocellular carcinoma [61]. Otherwise a study declared that poorer OS of NSCLC patients were related to *UBE2C* overexpression [62]. Upregulation of *UBE2C*-mediated autophagy promoted NSCLC progression [63].

Ribonucleotide reductase regulatory subunit M2 (*RRM2*) is a significant enzyme in DNA replication. High expression of *RRM2* was uncovered in glioblastoma [64] with promoting tumorigenicity [65], prostate cancer [66], NSCLC [67], and breast cancer. Overexpression of *RRM2* was strongly associated with worse survival in breast cancer and increased expression was shown in tamoxifen resistant patients [68]. Recently, a study [69] showed that chemotherapy resistance was associated with RRM2/EGFR/AKT signaling pathway in NSCLC.

Cyclin A2 (*CCNA2*), belonging to cyclin family, is a regulator of cell cycle. It activates cyclin dependent kinase 2(CK2) and promotes transformation through G1/S and G2/M [70]. *CCNA2* was overexpressed in bladder cancer [71], ER+ breast cancer and related to tamoxifen resistance [72], hepatoma with promoting cell proliferation [73] and lung adenocarcinoma [74]. *CCNA2* might have prognostic value for progression free survival(PFS) and OS in patients with lung cancer [75].

As an integral part of the mitotic spindle assembly checkpoint, mitotic arrest defect 2 like 1 (*MAD2L1*) is manifestly enriched in the cell cycle pathway and ensures that all chromosomes are arranged correctly on the metaphase plate [76, 77]. The deletion of tumor suppressor *MAD2L1* could lead to premature degradation of cyclin B, mitosis failure in human cells and tumorigenesis [78]. *MAD2L1* is overexpressed in multiple cancerous, such as breast cancer [79, 80] and gastric cancer [81], and lung cancer [82]. Recent studies [83–85] demonstrated that the prognosis of SCLC patients with high *MAD2L1* expression is worse than that with low *MAD2L1* expression. It implies that *MAD2L1* may be a promising therapeutic target for SCLC.

The interaction between eight hub genes and anti-tumor drugs were analyzed for the better understanding of the possibility of these genes as promising therapeutic targets for SCLC. As a result, we discovered that multiple drugs could change the expression of these hub genes.

As shown in Fig 9A, Sulforafan and Irinotecan promoted the expression of *CDC20*, while Sunitinib, Methotrexate, Fluorouracil, Dasatinib and Oxaliplatin inhibited the expression of *CDC20*. This suggested that Sunitinib, Methotrexate, Fluorouracil, Dasatinib, and Oxaliplatin might be viewed as targeted drug for the treatment of SCLC patients with high expression of *CDC20*. Interestingly, the interaction between Cisplatin or Paclitaxel and *CDC20* were controversial, and the conclusions drawn in different studies were inconsistent. In the same way, Sunitinib, Nocodazole, Topotecan, Cyclosporine, Dasatinib, Etoposide, Fluorouracil, Irinotecan, Methotrexate, and Azathioprine might be viewed as targeted drugs for the treatment of SCLC patients with high expression of BUB1. (Fig 9B) Methotrexate, Oxaliplatin, Carboplatin, Teniposide, Sunitinib, Cyclosporine, Aurofusarin, Dasatinib, Daunorubicin, Dronabinol, Etoposide, Fluorouracil, and Irinotecan might be viewed as targeted drugs for the treatment of SCLC patients with high expression of TOP2A. (Fig 9C) Vincristine, Cyclosporine, Methotrexate, Oxaliplatin, Topotecan and Cytarabine might be viewed as targeted drugs for the treatment of SCLC patients with high expression of UBE2C. (Fig 9D) Methotrexate, Oxaliplatin, Topotecan, Dasatinib, Etoposide and Fluorouracil might be viewed as targeted drugs for the treatment of SCLC patients with high expression of BUB1B. (Fig 9E) Methotrexate, Oxaliplatin, Sunitinib, Bicalutamide, Afureserti, Azathioprine, Dasatinib, Hydroxyurea and Topotecan might be viewed as targeted drugs for the treatment of SCLC patients with high expression of RRM2. (Fig 9F) Camptothecin and Fenretinide might be viewed as targeted drugs for the treatment of SCLC patients with high expression of MAD2L1. (Fig 9H) Surprisingly, no confirmed one could be viewed as targeted drug for the treatment of SCLC patients with high expression of CCNA2. (Fig 9G) However, further experimental study, including *in vivo* and *in vitro* experiments and clinical studies, are needed to explore the relationship between these hub genes and the prognosis of SCLC patients, and whether SCLC patients can benefit from these drugs.

At present, some related studies on the SCLC core genes have been published. Liao et al screened five hub genes from four GEO datasets (GSE60052, GSE43346, GSE15240 and GSE6044) by the raw data analysis by R software, functional annotation and pathway enrichment analysis, PPI network analysis, enrichment analyzes of two significant modules, and analysis of the expression levels of hub genes in the Oncomine database [84]. Wen et al discerned 10 hub genes from two GEO databases (GSE6044 and GSE11969) by using GEO2R tool, GO functional annotation and KEGG pathway enrichment analysis, PPI network, module analysis, hub genes selection, and validation of the mRNA expression levels of hub genes in the Oncomine database [86]. Mao et al identified 19 hub genes and 32 miRNAs from two GEO datasets (GSE6044 and GSE19945). Further analysis performed by functional annotation and pathway enrichment analysis of DEGs, PPI network, module analysis, hub genes screening, and miRNA-gene regulatory network [87]. Compared with the published literatures, we all found DEGs at RNA level based on GEO database and analyzed by bioinformatics methods. We constructed functional annotation and pathway enrichment analysis, PPI network, enrichment analyzes of significant modules, and verification of the expression levels of hub genes in the Oncomine database of DEGs by using similar methods. But we excluded two GEO datasets, including GSE6044 and GSE11969 which were in above published reports. The reasons are as follows: As reported, the human genome contains about 20,000 to 4,5000 genes encoding proteins [88]. But the number of probes in GSE6044 was only 8,793 genes, a lot less than 20,000~50,000 genes in that three GEO datasets. Therefore, the inadequacy of these data may lead to incomplete analysis. Secondly, the database related technology platform was applied 10 more years ago and had not been updated recently, therefore GSE6044 was ruled out. For GSE11969, all expression data with log_2_ (fold change) is less than 2, which is completely different from other GEO data sets. It may be unreasonable. Therefore, GSE11969 was excluded. More advantages of this study were as follows: Firstly, RT-qPCR were performed to validate the mRNA expression levels of hub genes by human samples. Secondly, this study screened potential prognostic biomarkers or therapeutic targets in SCLC and performed hub gene-drug interaction network analysis.

In the present paper, we have discussed that the overexpression of eight hub genes was closely related to the occurrence and development of SCLC, indicating that these hub genes might be acted as promising prognostic markers or therapeutic targets for SCLC. But our research also has limitations. Firstly, the data utilized in this study were all collected from public databases, but the quality of the data cannot be evaluated. Secondly, the sample capacity of relevant data is comparatively small. Thirdly, our study focused only on genes that changed significantly in multiple data sets, the characteristics of race, region, gender, age, tumor classification, stage, and smoking status were not considered integrally. Therefore, a lot of valuable biological information may be ignored in our research. Finally, as results, all eight hub genes were overexpressed in SCLC, but the corresponding mechanism has not been fully elucidated. Therefore, more molecular evidence is needed. Moreover, the current research in SCLC lacked prognostic data related to these hub genes, such as survival curves, which brought limitations to the clinical application value of hub genes. In this paper, the expression levels of eight hub genes were mainly analyzed. Whether these hub genes could be used as biomarkers or therapeutic targets of SCLC required further study.

## Conclusions

In conclusion, our bioinformatics analysis identified 208 DEGs, eight hub genes (*CDC20*, *BUB1*, *TOP2A*, *RRM2*, *CCNA2*, *UBE2C*, *MAD2L1*), and the mitotic cell cycle pathway that might play an momentous role in the development and prognosis of SCLC. As shown in database analysis and confirmed by human samples, overexpression of these hub genes indicated a poor prognosis for patients with SCLC. These results indicated that a comprehensive study of these DGEs will help us to understand the pathogenesis and progression of SCLC. However, since this study is mainly based on data analysis, further basic mechanism studies and clinical studies are needed to confirm these hypotheses in SCLC. We hope that this study can furnish certain new genomic basis for the individualized treatment of SCLC.

## Supporting information

S1 Table Gene expression microarray datasets for SCLC in GEO datasets, including GSE40275, GSE99316, and GSE60052. (XLSX)

S2 Table Information for the DEGs identified from the GEO datasets (|log2FC| ≥ 1, adjust *P* value < 0.05). (XLSX)

S3 Table Enrichment analysis of the DEGs. (XLSX)

S4 Table PPI network analysis of the DEGs. (XLSX)

S5 Table Pathway enrichment analysis of the DEGs in the two modules. (XLSX)

## References

[1] Bray F, Ferlay J, Soerjomataram I, Siegel RL, Torre LA, Jemal A. Global cancer statistics 2018: GLOBOCAN estimates of incidence and mortality worldwide for 36 cancers in 185 countries. CA Cancer J Clin. 2018;68(6):394–424. 10.3322/caac.21492 ; 30207593

[2] Xie D, Marks R, Zhang M, Jiang G, Jatoi A, Garces YI, et al Nomograms Predict Overall Survival for Patients with Small-Cell Lung Cancer Incorporating Pretreatment Peripheral Blood Markers. J Thorac Oncol. 2015;10(8):1213–20. 10.1097/JTO.0000000000000585 ; 26200277

[3] Antonia SJ, López-Martin JA, Bendell J, Ott PA, Taylor M, Eder JP, et al Nivolumab alone and nivolumab plus ipilimumab in recurrent small-cell lung cancer (CheckMate 032): a multicentre, open-label, phase 1/2 trial. Lancet Oncol. 2016;17(7):883–95. 10.1016/S1470-2045(16)30098-5 ; 27269741

[4] Reck M, Luft A, Szczesna A, Havel L, Kim SW, Akerley W, et al Phase III Randomized Trial of Ipilimumab Plus Etoposide and Platinum Versus Placebo Plus Etoposide and Platinum in Extensive-Stage Small-Cell Lung Cancer. J Clin Oncol. 2016;34(31):3740–8. 10.1200/JCO.2016.67.6601 ; 27458307

[5] Waqar SN, Morgensztern D. Treatment advances in small cell lung cancer (SCLC). Pharmacol Ther. 2017;180:16–23. 10.1016/j.pharmthera.2017.06.002 ; 28579387

[6] Sos ML, Dietlein F, Peifer M, Schöttle J, Balke-Want H, Müller C, et al A framework for identification of actionable cancer genome dependencies in small cell lung cancer. Proc Natl Acad Sci U S A. 2012;109(42):17034–9. 10.1073/pnas.1207310109 ; 23035247 PMC3479457

[7] Ferone G, Song JY, Krijgsman O, van der Vliet J, Cozijnsen M, Semenova EA, et al FGFR1 Oncogenic Activation Reveals an Alternative Cell of Origin of SCLC in Rb1/p53 Mice. Cell Rep. 2020;30(11):3837–50.e3. 10.1016/j.celrep.2020.02.052 ; 32187553 PMC7090386

[8] Peifer M, Fernández-Cuesta L, Sos ML, George J, Seidel D, Kasper LH, et al Integrative genome analyses identify key somatic driver mutations of small-cell lung cancer. Nat Genet. 2012;44(10):1104–10. 10.1038/ng.2396 ; 22941188 PMC4915822

[9] Wagner AH, Devarakonda S, Skidmore ZL, Krysiak K, Ramu A, Trani L, et al Recurrent WNT pathway alterations are frequent in relapsed small cell lung cancer. Nat Commun. 2018;9(1):3787 10.1038/s41467-018-06162-9 ; 30224629 PMC6141466

[10] Ni M, Liu X, Wu J, Zhang D, Tian J, Wang T, et al Identification of Candidate Biomarkers Correlated With the Pathogenesis and Prognosis of Non-small Cell Lung Cancer via Integrated Bioinformatics Analysis. Front Genet. 2018;9:469 10.3389/fgene.2018.00469 ; 30369945 PMC6194157

[11] Li S, Teng S, Xu J, Su G, Zhang Y, Zhao J, et al Microarray is an efficient tool for circRNA profiling. Brief Bioinform. 2019;20(4):1420–33. 10.1093/bib/bby006 ; 29415187

[12] Xu M, Gong S, Li Y, Zhou J, Du J, Yang C, et al Identifying Long Non-coding RNA of Prostate Cancer Associated With Radioresponse by Comprehensive Bioinformatics Analysis. Front Oncol. 2020;10:498 10.3389/fonc.2020.00498 ; 32318351 PMC7154134

[13] Ritchie ME, Phipson B, Wu D, Hu Y, Law CW, Shi W, et al limma powers differential expression analyses for RNA-sequencing and microarray studies. Nucleic acids research. 2015;43(7):e47 10.1093/nar/gkv007 ; 25605792 PMC4402510

[14] Pathan M, Keerthikumar S, Chisanga D, Alessandro R, Ang C-S, Askenase P, et al A novel community driven software for functional enrichment analysis of extracellular vesicles data. Journal of Extracellular Vesicles. 2017;6(1):1321455 10.1080/20013078.2017.1321455 . 28717418 PMC5505018

[15] Krzywinski M, Schein J, Birol I, Connors J, Gascoyne R, Horsman D, et al Circos: an information aesthetic for comparative genomics. Genome research. 2009;19(9):1639–45. 10.1101/gr.092759.109 ; 19541911 PMC2752132

[16] Szklarczyk D, Morris JH, Cook H, Kuhn M, Wyder S, Simonovic M, et al The STRING database in 2017: quality-controlled protein-protein association networks, made broadly accessible. Nucleic acids research. 2017;45(D1):D362–d8. 10.1093/nar/gkw937 ; 27924014 PMC5210637

[17] Shannon P, Markiel A, Ozier O, Baliga NS, Wang JT, Ramage D, et al Cytoscape: a software environment for integrated models of biomolecular interaction networks. Genome research. 2003;13(11):2498–504. 10.1101/gr.1239303 ; 14597658 PMC403769

[18] Bader GD, Hogue CW. An automated method for finding molecular complexes in large protein interaction networks. BMC bioinformatics. 2003;4:2 10.1186/1471-2105-4-2 ; 12525261 PMC149346

[19] Rhodes DR, Kalyana-Sundaram S, Mahavisno V, Varambally R, Yu J, Briggs BB, et al Oncomine 3.0: genes, pathways, and networks in a collection of 18,000 cancer gene expression profiles. Neoplasia. 2007;9(2):166–80. 10.1593/neo.07112 ; 17356713 PMC1813932

[20] Garber ME, Troyanskaya OG, Schluens K, Petersen S, Thaesler Z, Pacyna-Gengelbach M, et al Diversity of gene expression in adenocarcinoma of the lung. Proc Natl Acad Sci U S A. 2001;98(24):13784–9. 10.1073/pnas.241500798 ; 11707590 PMC61119

[21] Bhattacharjee A, Richards WG, Staunton J, Li C, Monti S, Vasa P, et al Classification of human lung carcinomas by mRNA expression profiling reveals distinct adenocarcinoma subclasses. Proc Natl Acad Sci U S A. 2001;98(24):13790–5. 10.1073/pnas.191502998 ; 11707567 PMC61120

[22] Davis AP, Grondin CJ, Johnson RJ, Sciaky D, McMorran R, Wiegers J, et al The Comparative Toxicogenomics Database: update 2019. Nucleic acids research. 2019;47(D1):D948–D54. 10.1093/nar/gky868 ; 30247620 PMC6323936

[23] Huang Y, Qi H, Zhang Z, Wang E, Yun H, Yan H, et al Gut REG3γ-Associated Lactobacillus Induces Anti-inflammatory Macrophages to Maintain Adipose Tissue Homeostasis. Front Immunol. 2017;8:1063 10.3389/fimmu.2017.01063 ; 28928739 PMC5591335

[24] Livak KJ, Schmittgen TD. Analysis of relative gene expression data using real-time quantitative PCR and the 2(-Delta Delta C(T)) Method. Methods. 2001;25(4):402–8. 10.1006/meth.2001.1262 ; 11846609

[25] Lynch TJ, Bell DW, Sordella R, Gurubhagavatula S, Okimoto RA, Brannigan BW, et al Activating mutations in the epidermal growth factor receptor underlying responsiveness of non-small-cell lung cancer to gefitinib. N Engl J Med. 2004;350(21):2129–39. 10.1056/NEJMoa040938 ; 15118073

[26] Soda M, Choi YL, Enomoto M, Takada S, Yamashita Y, Ishikawa S, et al Identification of the transforming EML4-ALK fusion gene in non-small-cell lung cancer. Nature. 2007;448(7153):561–6. 10.1038/nature05945 ; 17625570

[27] Bergethon K, Shaw AT, Ou SH, Katayama R, Lovly CM, McDonald NT, et al ROS1 rearrangements define a unique molecular class of lung cancers. J Clin Oncol. 2012;30(8):863–70. 10.1200/JCO.2011.35.6345 ; 22215748 PMC3295572

[28] Jänne PA, Meyerson M. ROS1 rearrangements in lung cancer: a new genomic subset of lung adenocarcinoma. J Clin Oncol. 2012;30(8):878–9. 10.1200/JCO.2011.39.4197 ; 22215755

[29] Testa U, Castelli G, Pelosi E. Lung Cancers: Molecular Characterization, Clonal Heterogeneity and Evolution, and Cancer Stem Cells. Cancers (Basel). 2018;10(8). 10.3390/cancers10080248 ; 30060526 PMC6116004

[30] Ma T, Liang F, Oesterreich S, Tseng GC. A Joint Bayesian Model for Integrating Microarray and RNA Sequencing Transcriptomic Data. J Comput Biol. 2017;24(7):647–62. 10.1089/cmb.2017.0056 ; 28541721 PMC5510692

[31] Li G, Su Q, Liu GQ, Gong L, Zhang W, Zhu SJ, et al Skewed X chromosome inactivation of blood cells is associated with early development of lung cancer in females. Oncol Rep. 2006;16(4):859–64. ; 16969506

[32] Zhou W, Machiela MJ, Freedman ND, Rothman N, Malats N, Dagnall C, et al Mosaic loss of chromosome Y is associated with common variation near TCL1A. Nat Genet. 2016;48(5):563–8. 10.1038/ng.3545 ; 27064253 PMC4848121

[33] Hanahan D, Weinberg RA. Hallmarks of cancer: the next generation. Cell. 2011;144(5):646–74. 10.1016/j.cell.2011.02.013 ; 21376230

[34] Gayyed MF, El-Maqsoud NM, Tawfiek ER, El Gelany SA, Rahman MF. A comprehensive analysis of CDC20 overexpression in common malignant tumors from multiple organs: its correlation with tumor grade and stage. Tumour Biol. 2016;37(1):749–62. 10.1007/s13277-015-3808-1 ; 26245990

[35] Kato T, Daigo Y, Aragaki M, Ishikawa K, Sato M, Kaji M. Overexpression of CDC20 predicts poor prognosis in primary non-small cell lung cancer patients. J Surg Oncol. 2012;106(4):423–30. 10.1002/jso.23109 ; 22488197

[36] Kidokoro T, Tanikawa C, Furukawa Y, Katagiri T, Nakamura Y, Matsuda K. CDC20, a potential cancer therapeutic target, is negatively regulated by p53. Oncogene. 2008;27(11):1562–71. 10.1038/sj.onc.1210799 ; 17873905

[37] Lee JH, Berger JM. Cell Cycle-Dependent Control and Roles of DNA Topoisomerase II. Genes (Basel). 2019;10(11). 10.3390/genes10110859 ; 31671531 PMC6896119

[38] Coss A, Tosetto M, Fox EJ, Sapetto-Rebow B, Gorman S, Kennedy BN, et al Increased topoisomerase IIalpha expression in colorectal cancer is associated with advanced disease and chemotherapeutic resistance via inhibition of apoptosis. Cancer Lett. 2009;276(2):228–38. 10.1016/j.canlet.2008.11.018 ; 19111388

[39] Winther TL, Torp SH. DNA topoisomerase IIα and mitosin expression predict meningioma recurrence better than histopathological grade and MIB-1 after initial surgery. PLoS One. 2017;12(3):e0172316 10.1371/journal.pone.0172316 ; 28301542 PMC5354255

[40] O’Malley FP, Chia S, Tu D, Shepherd LE, Levine MN, Bramwell VH, et al Topoisomerase II alpha and responsiveness of breast cancer to adjuvant chemotherapy. J Natl Cancer Inst. 2009;101(9):644–50. 10.1093/jnci/djp067 ; 19401546 PMC2677575

[41] Jain M, Zhang L, He M, Zhang YQ, Shen M, Kebebew E. TOP2A is overexpressed and is a therapeutic target for adrenocortical carcinoma. Endocr Relat Cancer. 2013;20(3):361–70. 10.1530/ERC-12-0403 ; 23533247 PMC4990817

[42] Han Z, Zhang M, Zhang Y, Yun W, Du X, Oncology DO. Overexpression of TOP2A in non- small cell lung cancer promotes cancer cell proliferation and invasion. Journal of Modern Oncology. 2016.

[43] Miura Y, Kaira K, Sakurai R, Sunaga N, Saito R, Hisada T, et al High expression of topoisomerase-II predicts favorable clinical outcomes in patients with relapsed small cell lung cancers receiving amrubicin. Lung Cancer. 2018;115:42–8. 10.1016/j.lungcan.2017.11.010 ; 29290260

[44] von Pawel J, Jotte R, Spigel DR, O’Brien ME, Socinski MA, Mezger J, et al Randomized phase III trial of amrubicin versus topotecan as second-line treatment for patients with small-cell lung cancer. J Clin Oncol. 2014;32(35):4012–9. 10.1200/JCO.2013.54.5392 ; 25385727

[45] Chang SM, Christian W, Wu MH, Chen TL, Lin YW, Suen CS, et al Novel indolizino [8,7-b]indole hybrids as anti-small cell lung cancer agents: Regioselective modulation of topoisomerase II inhibitory and DNA crosslinking activities. Eur J Med Chem. 2017;127:235–49. 10.1016/j.ejmech.2016.12.046 ; 28064078

[46] Feldhoff PW, Mirski SE, Cole SP, Sullivan DM. Altered subcellular distribution of topoisomerase II alpha in a drug-resistant human small cell lung cancer cell line. Cancer Res. 1994;54(3):756–62. ; 8306338

[47] Campling B, Baer K, Gerlach J, Lam Y, Cole S, Mirski S. Topoisomerase II levels and drug response in small cell lung cancer. Int J Oncol. 1997;10(5):885–93. 10.3892/ijo.10.5.885 ; 21533458

[48] Bolanos-Garcia VM, Blundell TL. BUB1 and BUBR1: multifaceted kinases of the cell cycle. Trends Biochem Sci. 2011;36(3):141–50. 10.1016/j.tibs.2010.08.004 ; 20888775 PMC3061984

[49] Zhang G, Kruse T, López-Méndez B, Sylvestersen KB, Garvanska DH, Schopper S, et al Bub1 positions Mad1 close to KNL1 MELT repeats to promote checkpoint signalling. Nat Commun. 2017;8:15822 10.1038/ncomms15822 ; 28604727 PMC5472792

[50] Li F, Kim H, Ji Z, Zhang T, Chen B, Ge Y, et al The BUB3-BUB1 Complex Promotes Telomere DNA Replication. Mol Cell. 2018;70(3):395–407.e4. 10.1016/j.molcel.2018.03.032 ; 29727616 PMC5982595

[51] Raaijmakers JA, van Heesbeen R, Blomen VA, Janssen LME, van Diemen F, Brummelkamp TR, et al BUB1 Is Essential for the Viability of Human Cells in which the Spindle Assembly Checkpoint Is Compromised. Cell Rep. 2018;22(6):1424–38. 10.1016/j.celrep.2018.01.034 ; 29425499

[52] Kapanidou M, Lee S, Bolanos-Garcia VM. BubR1 kinase: protection against aneuploidy and premature aging. Trends Mol Med. 2015;21(6):364–72. 10.1016/j.molmed.2015.04.003 ; 25964054

[53] Baker DJ, Dawlaty MM, Wijshake T, Jeganathan KB, Malureanu L, van Ree JH, et al Increased expression of BubR1 protects against aneuploidy and cancer and extends healthy lifespan. Nat Cell Biol. 2013;15(1):96–102. 10.1038/ncb2643 ; 23242215 PMC3707109

[54] Ding Y, Hubert CG, Herman J, Corrin P, Toledo CM, Skutt-Kakaria K, et al Cancer-Specific requirement for BUB1B/BUBR1 in human brain tumor isolates and genetically transformed cells. Cancer Discov. 2013;3(2):198–211. 10.1158/2159-8290.CD-12-0353 ; 23154965 PMC3632446

[55] Lee E, Pain M, Wang H, Herman JA, Toledo CM, DeLuca JG, et al Sensitivity to BUB1B Inhibition Defines an Alternative Classification of Glioblastoma. Cancer Res. 2017;77(20):5518–29. 10.1158/0008-5472.CAN-17-0736 ; 28855212 PMC5645262

[56] Jaffrey RG, Pritchard SC, Clark C, Murray GI, Cassidy J, Kerr KM, et al Genomic instability at the BUB1 locus in colorectal cancer, but not in non-small cell lung cancer. Cancer Res. 2000;60(16):4349–52. ; 10969775

[57] Haruki N, Saito H, Harano T, Nomoto S, Takahashi T, Osada H, et al Molecular analysis of the mitotic checkpoint genes BUB1, BUBR1 and BUB3 in human lung cancers. Cancer Lett. 2001;162(2):201–5. 10.1016/s0304-3835(00)00675-3 ; 11146226

[58] Loussouarn D, Campion L, Leclair F, Campone M, Charbonnel C, Ricolleau G, et al Validation of UBE2C protein as a prognostic marker in node-positive breast cancer. Br J Cancer. 2009;101(1):166–73. 10.1038/sj.bjc.6605122 ; 19513072 PMC2713693

[59] Chen S, Chen Y, Hu C, Jing H, Cao Y, Liu X. Association of clinicopathological features with UbcH10 expression in colorectal cancer. J Cancer Res Clin Oncol. 2010;136(3):419–26. 10.1007/s00432-009-0672-7 ; 19779934 PMC11827792

[60] Liu G, Zhao J, Pan B, Ma G, Liu L. UBE2C overexpression in melanoma and its essential role in G2/M transition. J Cancer. 2019;10(10):2176–84. 10.7150/jca.32731 ; 31258721 PMC6584412

[61] Ieta K, Ojima E, Tanaka F, Nakamura Y, Haraguchi N, Mimori K, et al Identification of overexpressed genes in hepatocellular carcinoma, with special reference to ubiquitin-conjugating enzyme E2C gene expression. Int J Cancer. 2007;121(1):33–8. 10.1002/ijc.22605 ; 17354233

[62] Zhang Z, Liu P, Wang J, Gong T, Zhang F, Ma J, et al Ubiquitin-conjugating enzyme E2C regulates apoptosis-dependent tumor progression of non-small cell lung cancer via ERK pathway. Med Oncol. 2015;32(5):149 10.1007/s12032-015-0609-8 ; 25832867

[63] Guo J, Wu Y, Du J, Yang L, Chen W, Gong K, et al Deregulation of UBE2C-mediated autophagy repression aggravates NSCLC progression. Oncogenesis. 2018;7(6):49 10.1038/s41389-018-0054-6 ; 29904125 PMC6002383

[64] Li C, Zheng J, Chen S, Huang B, Li G, Feng Z, et al RRM2 promotes the progression of human glioblastoma. J Cell Physiol. 2018;233(10):6759–67. 10.1002/jcp.26529 ; 29667764

[65] Rasmussen RD, Gajjar MK, Tuckova L, Jensen KE, Maya-Mendoza A, Holst CB, et al BRCA1-regulated RRM2 expression protects glioblastoma cells from endogenous replication stress and promotes tumorigenicity. Nat Commun. 2016;7:13398 10.1038/ncomms13398 ; 27845331 PMC5116074

[66] Mazzu YZ, Armenia J, Chakraborty G, Yoshikawa Y, Coggins SA, Nandakumar S, et al A Novel Mechanism Driving Poor-Prognosis Prostate Cancer: Overexpression of the DNA Repair Gene, Ribonucleotide Reductase Small Subunit M2 (RRM2). Clin Cancer Res. 2019;25(14):4480–92. 10.1158/1078-0432.CCR-18-4046 ; 30996073 PMC6820162

[67] Yang Y, Li S, Cao J, Li Y, Hu H, Wu Z. RRM2 Regulated By LINC00667/miR-143-3p Signal Is Responsible For Non-Small Cell Lung Cancer Cell Progression. Onco Targets Ther. 2019;12:9927–39. 10.2147/OTT.S221339 ; 31819489 PMC6876211

[68] Putluri N, Maity S, Kommagani R, Creighton CJ, Putluri V, Chen F, et al Pathway-centric integrative analysis identifies RRM2 as a prognostic marker in breast cancer associated with poor survival and tamoxifen resistance. Neoplasia. 2014;16(5):390–402. 10.1016/j.neo.2014.05.007 ; 25016594 PMC4198742

[69] Huang N, Guo W, Ren K, Li W, Jiang Y, Sun J, et al LncRNA AFAP1-AS1 Supresses miR-139-5p and Promotes Cell Proliferation and Chemotherapy Resistance of Non-small Cell Lung Cancer by Competitively Upregulating RRM2. Front Oncol. 2019;9:1103 10.3389/fonc.2019.01103 ; 31696057 PMC6817562

[70] Blanchard JM. Cyclin A2 transcriptional regulation: modulation of cell cycle control at the G1/S transition by peripheral cues. Biochem Pharmacol. 2000;60(8):1179–84. 10.1016/s0006-2952(00)00384-1 ; 11007956

[71] Li J, Ying Y, Xie H, Jin K, Yan H, Wang S, et al Dual regulatory role of CCNA2 in modulating CDK6 and MET-mediated cell-cycle pathway and EMT progression is blocked by miR-381-3p in bladder cancer. Faseb j. 2019;33(1):1374–88. 10.1096/fj.201800667R ; 30138038

[72] Gao T, Han Y, Yu L, Ao S, Li Z, Ji J. CCNA2 is a prognostic biomarker for ER+ breast cancer and tamoxifen resistance. PLoS One. 2014;9(3):e91771 10.1371/journal.pone.0091771 ; 24622579 PMC3951414

[73] Yang F, Gong J, Wang G, Chen P, Yang L, Wang Z. Waltonitone inhibits proliferation of hepatoma cells and tumorigenesis via FXR-miR-22-CCNA2 signaling pathway. Oncotarget. 2016;7(46):75165–75. 10.18632/oncotarget.12614 ; 27738335 PMC5342731

[74] Li J, Liu L, Liu X, Xu P, Hu Q, Yu Y. The Role of Upregulated DDX11 as A Potential Prognostic and Diagnostic Biomarker in Lung Adenocarcinoma. J Cancer. 2019;10(18):4208–16. 10.7150/jca.33457 ; 31413739 PMC6691710

[75] Brcic L, Heidinger M, Sever AZ, Zacharias M, Jakopovic M, Fediuk M, et al Prognostic value of cyclin A2 and B1 expression in lung carcinoids. Pathology. 2019;51(5):481–6. 10.1016/j.pathol.2019.03.011 ; 31230818

[76] Abal M, Obrador-Hevia A, Janssen KP, Casadome L, Menendez M, Carpentier S, et al APC inactivation associates with abnormal mitosis completion and concomitant BUB1B/MAD2L1 up-regulation. Gastroenterology. 2007;132(7):2448–58. 10.1053/j.gastro.2007.03.027 ; 17570218

[77] Krishnan R, Goodman B, Jin DY, Jeang KT, Collins C, Stetten G, et al Map location and gene structure of the Homo sapiens mitotic arrest deficient 2 (MAD2L1) gene at 4q27. Genomics. 1998;49(3):475–8. 10.1006/geno.1998.5233 ; 9615237

[78] Michel L, Diaz-Rodriguez E, Narayan G, Hernando E, Murty VV, Benezra R. Complete loss of the tumor suppressor MAD2 causes premature cyclin B degradation and mitotic failure in human somatic cells. Proc Natl Acad Sci U S A. 2004;101(13):4459–64. 10.1073/pnas.0306069101 ; 15070740 PMC384769

[79] Qi L, Zhou B, Chen J, Hu W, Bai R, Ye C, et al Significant prognostic values of differentially expressed-aberrantly methylated hub genes in breast cancer. J Cancer. 2019;10(26):6618–34. 10.7150/jca.33433 ; 31777591 PMC6856906

[80] Scintu M, Vitale R, Prencipe M, Gallo AP, Bonghi L, Valori VM, et al Genomic instability and increased expression of BUB1B and MAD2L1 genes in ductal breast carcinoma. Cancer Lett. 2007;254(2):298–307. 10.1016/j.canlet.2007.03.021 ; 17498870

[81] Bargiela-Iparraguirre J, Prado-Marchal L, Pajuelo-Lozano N, Jiménez B, Perona R, Sánchez-Pérez I. Mad2 and BubR1 modulates tumourigenesis and paclitaxel response in MKN45 gastric cancer cells. Cell Cycle. 2014;13(22):3590–601. 10.4161/15384101.2014.962952 ; 25483095 PMC4615044

[82] Kato T, Daigo Y, Aragaki M, Ishikawa K, Sato M, Kondo S, et al Overexpression of MAD2 predicts clinical outcome in primary lung cancer patients. Lung Cancer. 2011;74(1):124–31. 10.1016/j.lungcan.2011.01.025 ; 21376419

[83] Wu Y, Tan L, Chen J, Li H, Ying H, Jiang Y, et al MAD2 Combined with Mitotic Spindle Apparatus (MSA) and Anticentromere Antibody (ACA) for Diagnosis of Small Cell Lung Cancer (SCLC). Med Sci Monit. 2018;24:7541–7. 10.12659/MSM.909772 ; 30346937 PMC6354645

[84] Liao Y, Yin G, Wang X, Zhong P, Fan X, Huang C. Identification of candidate genes associated with the pathogenesis of small cell lung cancer via integrated bioinformatics analysis. Oncol Lett. 2019;18(4):3723–33. 10.3892/ol.2019.10685 ; 31516585 PMC6732946

[85] Ni Z, Wang X, Zhang T, Li L, Li J. Comprehensive analysis of differential expression profiles reveals potential biomarkers associated with the cell cycle and regulated by p53 in human small cell lung cancer. Exp Ther Med. 2018;15(4):3273–82. 10.3892/etm.2018.5833 ; 29545845 PMC5841087

[86] Wen P, Chidanguro T, Shi Z, Gu H, Wang N, Wang T, et al Identification of candidate biomarkers and pathways associated with SCLC by bioinformatics analysis. Mol Med Rep. 2018;18(2):1538–50. 10.3892/mmr.2018.9095 ; 29845250 PMC6072191

[87] Mao Y, Xue P, Li L, Xu P, Cai Y, Chu X, et al Bioinformatics analysis of mRNA and miRNA microarray to identify the key miRNA-gene pairs in small-cell lung cancer. Mol Med Rep. 2019;20(3):2199–208. 10.3892/mmr.2019.10441 ; 31257520 PMC6691276

[88] Consortium E. The ENCODE (ENCyclopedia of DNA elements) Project Science. 2004;306(5696):636–40. 10.1126/science.1105136 15499007

