# Supplementary material for: Identification of potential target genes and crucial pathways in small cell lung cancer based on bioinformatic strategy and human samples
Source: PLoS One. 2020 Nov 13;15(11):e0242194. doi: 10.1371/journal.pone.0242194 (PMC7665632; doi:10.1371/journal.pone.0242194)
Supplement: Attachment — Submitted filename: Response to Reviewers.docx [file pone.0242194.s006.docx]

Dear editors：

On behalf of my co-author, we thank you very much for giving us an opportunity to revise our manuscript, we greatly appreciate editor and reviewers for their positive and constructive comments and suggestions on our manuscript entitled "Identification of potential target genes and crucial pathways in small cell lung cancer based on bioinformatic strategy and human samples" (the title has been renewed; Paper ID : PONE-D-20-29317). We have finished the proof reading and checking carefully, and some corrections about the proof and answers to the questions are provided below.

**Corrections:**

(1) The manuscript was revised by us from the title to the " References " and made according to the style requirements of PLOS ONE.

Moreover, Dr. Su was asked to rewrite the manuscript word by word. The revised manuscript is attached. Detailed information of Dr. Su:

Name: Xiao-min Su,

E-mail: [xiaominsu@nankai.edu.cn](mailto:xiaominsu@nankai.edu.cn);

Affiliated institution: Department of Immunology, Nankai University School of Medicine, Nankai University, Tianjin 300071, China.

Address: No.94, Weijin Road, Nankai, Tianjin 300071, China.

Mobile phone: +86-18630934876

List of published articles in recent years:

1. Cao Shuisong#, Su Xiaomin#, Zeng Benhua#, et al. The Gut Epithelial Receptor LRRC19Promotes the Recruitment of Immune Cells and Gut Inflammation. Cell Rep, 2016, 14(4):695-707.

2. Su Xiaomin#, Yan Hui#, Huang Yugang#, et al. Expression of FABP4, adipsin and adiponectin in Paneth cells ismodulated by gut Lactobacillus. Sci Rep, 2015, 5:18588-18588.

3. Su Xiaomin#, Mei Shiyue, Liang Xue, et al. Epigenetically modulated LRRC33 actsas a negativephysiological regulator for multiple Toll-like receptors. J Leukoc Biol, 2014, 96(1):17-26.

4. Su Xiaomin#, Min Siping#, Cao Shuisong#, et al. LRRC19 expressed in the kidney

induces TRAF2/6-mediated signals to prevent infection by uropathogenicbacteria. Nat

Commun, 2014, 5:4434-4434.

(2) The title of manuscript has been renewed as "Identification of potential target genes and crucial pathways in small cell lung cancer based on bioinformatic strategy and human samples".

(3) The brief title of manuscript has been renewed as " Potential target genes and crucial pathway in SCLC".

(4) According to the Journal Requirements, we have corrected the naming of supporting information and manuscript.

(5) Ethical statements written in any section besides the " Materials and Methods "have been deleted.

**Answers to questions from reviewers:**

(1) Comments of reviewer #1: Huang et al. explored the potential pathogenic and prognostic crucial genes and key pathways of SCLC via bioinformatics analysis of public datasets. The authors identified eight hub genes that could be new biomarkers for prognosis and treatment for SCLC. Since there are several publications that similarly analyze and identify hub genes in SCLC, the authors need to discuss in depth about commonality and difference between the current and past analyses. The authors also need to discuss further the interpretation and significance of the Gene-Drug Interaction analysis in Fig. 9.

Answer to Reviewer #1:

Firstly, Due to the similar analysis and identification of hub genes in SCLC in some publications, we discuss the similarities and differences between current and past analyses. Please see the 432nd line to the 444th line of the revised manuscript.

Secondly, we further discussed the interpretation and significance of the gene drug interaction analysis in Fig. 9. Please see the 396th to 417th line of the revised manuscript.

(2) Minor Revisions 1: Figure 2. Legend. (F) should be capitalized.

Answer to Minor Revisions 1: In Figure 2 legend, (F) has been capitalized. Please see the 213rd line of the revised manuscript.

(3) Minor Revisions 2: Figure 3. The figure legend should be more detailed as to what the red and yellow lines are denoting. It will make the data descriptive and informative.

Answer to Minor Revisions 2: In Figure3, the figure legend has been detailed as to what the X axis, Y axis, red and yellow lines are denoting. Please see the 213rd line to the 216th line of the revised manuscript.

(4) Minor Revisions 3: The authors saw that the downregulated genes were mainly associated with immune signaling. The authors have not discussed this observation in the discussion section. It would be informative if the authors discussed about what is known and what are the implications.

Answer to Minor Revisions 3: This study found that the down regulated genes were mainly related to immune signals. We further discussed this observation in the discussion section. Please see the 318th line to the 322nd line of the revised manuscript

Lastly, we have upload figure files to the Preflight Analysis and Conversion Engine (PACE) digital diagnostic tool to ensure these figures meet PLOS requirements.

We deeply appreciate the efficient, professional and rapid processing of our paper by your team. If there is anything else wo should do, please don't hesitate to contact us at the address below.

Thank you and best regards.

Your sincerely,

Yu-gang Huang

Corresponding author:

Name: Yu-gang Huang; E-mail: [huangyg2018@outlook.com](mailto:huangyg2018@outlook.com)
